# Magnetoelectric microrobots for spinal cord injury regeneration

**DOI:** 10.1038/s41563-026-02625-3

**Published:** 2026-06-02

**Authors:** Hao Ye, Jingjing Zang, Jiawei Zhu, Denis von Arx, Jian Zhao, Vitaly Pustovalov, Minmin Mao, Qiao Tang, Andrea Veciana, Harun Torlakcik, Elric Zhang, Semih Sevim, Roger Sanchis-Gual, Quan Gao, Xiang-Zhong Chen, Daniel Ahmed, Maria V. Sanchez-Vives, Josep Puigmartí-Luis, Cong Luo, Bradley J. Nelson, Stephan C. F. Neuhauss, Salvador Pané

**Affiliations:** 1 Multi-Scale Robotics Lab (MSRL), Institute of Robotics & Intelligent Systems (IRIS), ETH Zurich, Zurich, Switzerland; 2 Department of Molecular Life Sciences, University of Zurich, Zurich, Switzerland; 3 Department of Pharmaceutics, Wuya College of Innovation, Shenyang Pharmaceutical University, Shenyang, China; 4 College of Electronic Information and Engineering, Hangzhou Dianzi University, Hangzhou, China; 5 Key Laboratory of Biomaterials of Guangdong Higher Education Institutes, Engineering Technology Research Center of Drug Carrier of Guangdong, Department of Biomedical Engineering, Jinan University, Guangzhou, China; 6 Instituto de Ciencia Molecular, Universitat de València, Paterna, Spain; 7 International Institute for Intelligent Nanorobots and Nanosystems, College of Intelligent Robotics and Advanced Manufacturing, State Key Laboratory of Photovoltaic Science and Technology, Shanghai Frontiers Science Research Base of Intelligent Optoelectronics and Perception, Institute of Optoelectronics, Fudan University, Shanghai, China; 8 Zhejiang Key Laboratory of Extreme Environment Functional Materials, Yiwu Research Institute of Fudan University, Yiwu, China; 9 Acoustics Robotics Systems Lab (ARSL), ETH-Zürich, Rüschlikon, Switzerland; 10 Acoustic Robotics Systems Lab, ARTORG Center for Biomedical Engineering Research, Faculty of Medicine, University of Bern, Bern, Switzerland; 11 Institut d’Investigacions Biomèdiques August Pi i Sunyer (IDIBAPS), Barcelona, Spain; 12 Institució Catalana de Recerca i Estudis Avançats (ICREA), Barcelona, Spain; 13 Departament de Ciència dels Materials i Química Física Institut de Química Teòrica i Computacional, University of Barcelona, Barcelona, Spain

**Keywords:** Neural stem cells, Regenerative medicine, Stem-cell research, Magnetic properties and materials, Tissue engineering and regenerative medicine

## Abstract

Spinal cord injury remains difficult to treat because of the intrinsically limited regenerative capacity of neurons. Although neural progenitor cell (NPC) therapies are promising, inadequate graft survival, uncontrolled differentiation and weak functional integration continue to restrict outcomes. Here we report biohybrid microrobots called NPCbots, fabricated by integrating human-induced pluripotent-stem-cell-derived NPCs with magnetoelectric nanoparticles, enabling wireless magnetic navigation and non-invasive neuronal stimulation. A lab-on-a-chip platform allows scalable fabrication and maintains cell viability and differentiation capacity. In a zebrafish spinal cord injury model, alternating magnetic field stimulation of NPCbots induced rapid in vivo neuronal and astrocytic differentiation, enhanced graft integration at the lesion site, and near-complete recovery of swimming and exploratory behaviours within 3 days. In a non-regenerating murine model of complete spinal cord transection, NPCbots were well tolerated for at least 28 days, localized effectively to the injury site, promoted neural differentiation and resulted in substantial improvements in motor function within 4 weeks. These results demonstrate that magnetically guided NPCbots combined with non-invasive magnetoelectric stimulation promote neural repair and functional recovery in preclinical spinal cord injury models.

## Abstract (web summary)

Microrobots made of human-induced pluripotent stem cells and magnetoelectric nanoparticles promote lesion repair and motor improvements after spinal cord injury in zebrafish and mice under magnetic guidance and stimulation.

## Main

Cell therapies represent a burgeoning segment of biotechnology, aimed at curing diseases by enhancing the localization of therapeutic cells within target tissues. These therapies are diverse, encompassing tissue regeneration, restoration of biological functions and enhancement of the body’s intrinsic disease-fighting capabilities^1–4^. Unlike many drugs and biological agents, delivered cells can traverse biological barriers; respond to and adapt to biological signals; and specifically target cells, tissues or organs^5–8^. Although the safety and efficacy of cell therapies have been enhanced by minimally invasive cell delivery techniques, therapeutic efficacy is still suboptimal, with low cell survival rates and unreliable in vivo differentiation into targeted cells^9–11^. For instance, selective colonization by neural stem cells, which aim to differentiate only at the target site can address these issues^12–16^. Neurotrophins, such as nerve growth factor, typically act as chemical stimuli^17^, but are less effective in vivo due to rapid diffusion rates, delivery challenges and short half-lives^18^. These limitations might be overcome by directly stimulating cells to initiate neuronal stimulation at specific post-delivery times^19,20^. Various external stimuli, including light^21^, magnetic fields^20,22^ and sound waves^23^, have been utilized to promote local neuronal differentiation. Among these, magnetic fields are well suited for deep tissue penetration with minimal attenuation due to the low magnetic susceptibility of biological materials^24–27^.

**Fig. 1:**
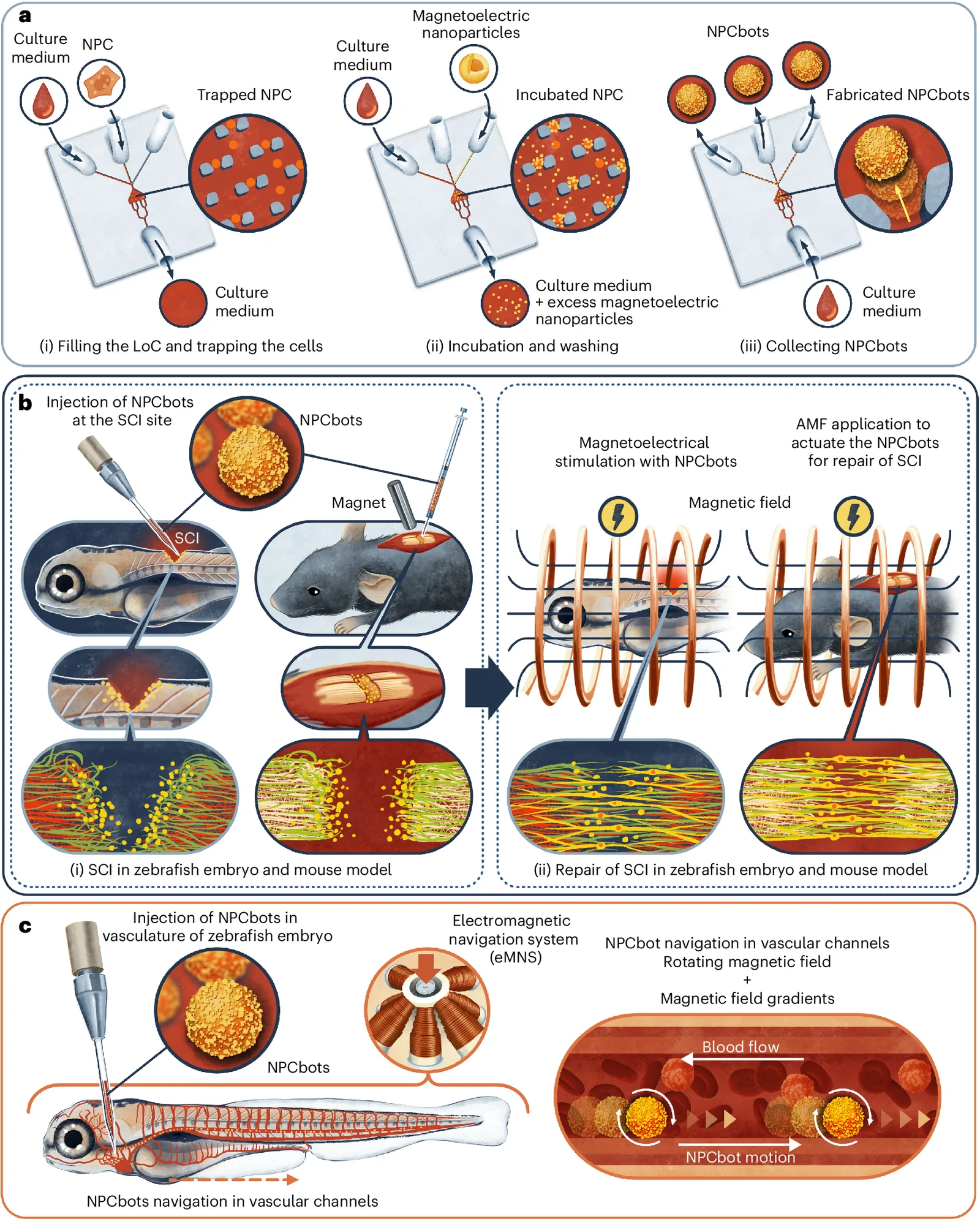
Schematic of NPCbot fabrication and application processes. **a**, Overview of the NPCbot’s fabrication steps, including NPC trapping, incubation of NPCs with CFO–BTO nanoparticles and collection of NPCbots. **b**, Following the injection of NPCbots, the application of an AMF to the SCI model in zebrafish larvae and mouse models enables the differentiation of NPCbots and helps bridge the injured neural connections. **c**, In vivo manipulation of the NPCbots within the DA of zebrafish larvae, depicting the NPCbots’ rolling motion through the targeted area within zebrafish larvae.

In recent years, the approach to treat spinal cord injuries (SCI) increasingly focused on the potential benefits of neural progenitor cell (NPC) transplants^28^. Despite considerable progress, obstacles related to graft survival, controlled differentiation and neural integration continue to severely constrain therapeutic successes and the attainment of real functional recovery^28–30^. Our study introduces NPC-based microrobots (NPCbots)—a novel class of biohybrid microrobots. The unique construction of CoFe_2_O_4_–BaTiO_3_ (CFO–BTO) nanoparticles, through cobalt ferrite (CFO) and barium titanate (BTO) synthesis, provides dual functionalities of magnetic manipulation and magnetoelectric stimulation^31–34^. This enhances both operational efficiency and therapeutic impact of the microrobots. Techniques such as high-angle annular dark-field scanning transmission electron microscopy (HAADF-STEM) and energy-dispersive X-ray (EDX) spectroscopy mapping, verified the integrity of the core–shell structure and effective magnetoelectric coupling. These CFO–BTO magnetoelectric core–shell nanoparticles were then integrated with human-induced pluripotent stem cell (iPSC)-derived NPCs, enabling in vivo magnetic navigation and specific neuronal differentiation within complex biological settings. The fabrication of NPCbots using a bidirectional continuous-flow microfluidic device facilitates their mass production (Fig. 1a).

Our approach is further illustrated by the deployment of these NPCbots within both zebrafish and non-regenerating murine model of SCI. By integrating magnetic guidance with in vivo NPC stimulation, we not only enhance neural repair capabilities but also markedly improve functional recovery, as evidenced through comprehensive behavioural and molecular assessments (Fig. 1b,c). The use of alternating magnetic fields (AMF) to stimulate the NPCbots results in marked neuronal and astrocytic differentiation, effectively leveraging the magnetoelectric properties of CFO–BTO nanoparticles to direct cellular behaviour and promote neural repair (Fig. 1b). This study contributes to the fields of nanotechnology and regenerative medicine by providing a potential approach to the treatment of neurodegenerative diseases. It introduces a platform that allows for microscale positioning-guided and efficient restoration of neural functions through non-invasive means. By utilizing microfluidic technologies—that is, lab-on-a-chip (LoC) devices—and magnetoelectric nanoparticles, NPCbots represent an advancement in therapeutic applications. They offer promising potential for improving medical treatments for SCI and related conditions.

## Microfluidics enables assembly of magnetoelectric NPCbots

LoC devices have realized the rapid and efficient mass production of biohybrid microrobots. In this investigation, we detail the fabrication process of these innovative microrobots (Fig. 1a). Each microrobot incorporates CFO–BTO and human iPSC-derived NPCs. The CFO–BTO nanoparticles are fabricated by synthesizing a magnetostrictive CFO core and encasing it in a piezoelectric BTO shell, where the magnetic-field-induced strain in CFO is converted into an electrical output by BTO, thereby enabling magnetoelectric coupling^33^. This configuration is specifically designed to facilitate magnetic manipulation and in vivo NPC magnetoelectric stimulation of the microrobot.

The synthesis of these CFO–BTO core–shell magnetoelectric nanoparticles begins with a co-precipitation process, followed by hydrothermal and sol–gel methods. Initially, cetyltrimethylammonium bromide is dissolved in deionized water, with subsequent additions of ferric chloride hexahydrate and cobalt(II) chloride. The formation of CFO nanoparticles is initiated by adding sodium hydroxide, followed by hydrothermal treatment in a sealed autoclave to enhance crystallinity. The BTO shell is then formed using a sol–gel technique, involving a precursor solution of barium carbonate, citric acid and titanium isopropoxide. This precursor is mixed with the CFO nanoparticles to form a gel, which is subsequently dried and annealed to complete the synthesis of the CFO–BTO core–shell nanoparticles.

**Fig. 2:**
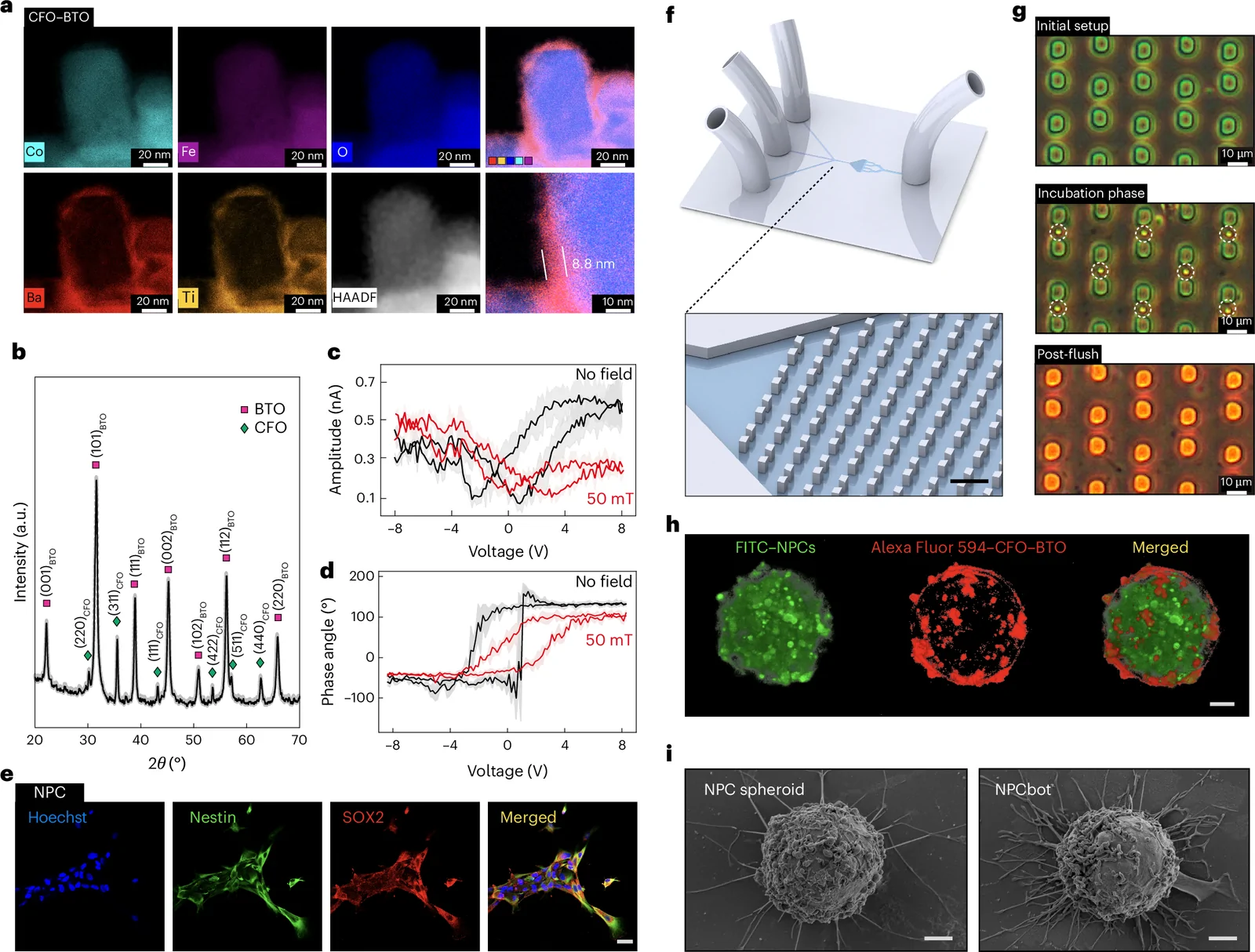
Fabrication and characterization of NPCbots. **a**, HAADF-STEM and EDX analyses of CFO–BTO core–shell nanoparticles. **b**, X-ray diffraction patterns confirming the crystalline structure of CFO–BTO powders. **c**,**d**, Magnetic-field-dependent local piezoresponse force microscopy hysteresis loops (amplitude (**c**) and phase (**d**)) from CFO–BTO core–shell nanoparticles, with (red) and without (black) an applied magnetic field of 50 mT (*n* = 3 experiments; data are presented as mean ± s.d.). **e**, Immunofluorescence staining for Nestin and SOX2 in NPCs, highlighting progenitor cell markers. Scale bar, 20 μm. **f**, LoC device design featuring a bidirectional continuous-flow microfluidic system that includes a large trapping chamber for cell capture and incubation; three inlets for introducing growth medium, cells and nanoparticles; and a single outlet that can also function as an inlet during the collection of NPCbots. **g**, Optical microscopy image documenting the fabrication process of NPCbots. **h**, Confocal microscopy images of NPCbots labelled with Alexa Fluor 594-conjugated CFO–BTO nanoparticles and FITC-labelled NPCs after 30 min of incubation. Scale bar, 2 μm. **i**, SEM images of an NPC spheroid and an assembled NPCbot, demonstrating the structural integrity and surface morphology. Scale bars, 2 μm. Source data

The characterization of these nanoparticles is achieved using HAADF-STEM coupled with EDX mapping. This confirms the core–shell structure, revealing a CFO core rich in Co, Fe and O, and a BTO shell containing Ba, Ti and O elements (Fig. 2a). The dimensions of the core and shell are 99.4 ± 4.2 nm and 7 ± 3.5 nm, respectively. X-ray diffraction analyses further confirm the phase purity and crystalline structure of the nanoparticles, which identified characteristic reflections corresponding to the ferrite CFO phase of the ${Fd}\bar{3}{m}$ space group (ICDD: 98-016-7445) and the ferroelectric BTO phase of the *P*4*mm* space group (ICDD: 98-001-5453)^35^, with no impurities or intermediate phases detected (Fig. 2b). Furthermore, the magnetoelectric coupling properties of the nanoparticles were explored by assessing the local piezoresponse under magnetic fields. The application of a 50-mT direct current (d.c.) in-plane magnetic field altered the local piezoelectric hysteresis loops, demonstrating magnetoelectric coupling in the CFO–BTO nanoparticles. This was evidenced by the notable shifts in both positive and negative coercive voltages (d*V* = 1.81 V) with respect to no field, suggesting that the strain generated within the CFO (magnetostrictive phase) effectively transferred to the BTO (piezoelectric phase), thereby facilitating polarization^33,36^. The observed asymmetry in the piezoresponse loops, evident from an offset under the magnetic influence, probably resulted from an electric field generated through the magnetoelectric effect, consistent with the strain-mediated magnetoelectric functionality within these core–shell nanoparticles (Fig. 2c,d).

The stemness of NPCs is critical for the fabrication of NPCbots. Nestin and SOX2 are extensively characterized as NPC markers and are not present in mature neurons^37^. Both markers colocalize in NPCs, confirming that the NPCs retain their stem-like properties without undergoing differentiation (Fig. 2e).

To construct the NPCbots, a bidirectional continuous-flow microfluidic device was utilized (Fig. 2f and Supplementary Fig. 1). This device is engineered with a large trapping chamber, specifically optimized for capturing and incubating cells to synthesize functional microrobots. It incorporates three inlets on one side for the introduction of growth medium, cells and nanoparticles, whereas a single outlet on the opposing side also functions inversely as an inlet during the collection phase of microrobots (Fig. 1a). The microfluidic trapping chamber is designed to have a triangular shape for two reasons: it maximizes the trapping efficiency based on the probabilistic distribution of cells within the fluid stream and facilitates an accelerated reverse flow during the collection phase, which is critical for preventing clogging at the chamber’s entrance. The traps, with a minimum gap of 6 µm, are finely tuned to ensnare a broad spectrum of cell sizes and minimize flow resistance that might allow cells to evade capture. The microfluidic chamber is designed with a height of 8–10 µm to prevent the simultaneous trapping of multiple cells at one site. During NPCbot fabrication (Fig. 1a), NPCs are ensnared within these traps via a controlled low-flow-rate microfluidic stream. This is followed by the introduction of CFO–BTO flow for in situ incubation within the device. After incubation, the outlet of the device is reconfigured to serve as an inlet, using a pump to facilitate a reverse flow that discharges the cultivated microrobots from their traps. The original inlets are then repurposed as outlets for microrobot collection. This innovative bidirectional design not only simplifies the trapping and release of microrobots but also supports their mass production, enhancing the efficiency and convenience of biohybrid microrobot fabrication (Fig. 2g).

The viability of the synthesized NPCbots was assessed by introducing varying concentrations of CFO–BTO nanoparticles, selecting a concentration of 33 mg ml^−1^ (1 ng of CFO–BTO per cell), which demonstrated over 85% viability (Supplementary Fig. 2). Further investigations explored the effects of incubation time on cellular uptake of CFO–BTO. To fluorescently label CFO–BTO, nanoparticles were initially coated with (3-aminopropyl)triethoxysilane (APTES), facilitating amino group attachment to their surfaces. Spectral analysis confirmed the presence of amine groups, as evidenced by the Si–O–Si signal derived from the CFO–BTO-conjugated APTES (Supplementary Fig. 3). Subsequently, these APTES-coated nanoparticles were incubated with Alexa Fluor 594 NHS ester (succinimidyl ester), which promoted the formation of stable amide bonds between the NHS ester and amine groups. After a 1-h incubation at 37 °C, significant cellular uptake by NPCs was observed, determining a 30-min incubation period as optimal for NPCbot fabrication to ensure the maximum interaction with the cell membrane, potentially influencing membrane channels (Supplementary Fig. 4).

Following the post-labelling of CFO–BTO with Alexa Fluor 594, there were negligible changes in the size and zeta potential of the nanoparticles (Supplementary Fig. 5), facilitating further exploration of their distribution. The NPCbots were subsequently stained using a live/dead staining kit, incorporating a live dye for visualization. Confocal microscopy enhanced by *z*-stack analysis documented the detailed distribution patterns of the labelled CFO–BTO on the NPC surface (Fig. 2h). Scanning electron microscopy (SEM) was used to assess the morphological differences between NPCbots and NPC spheroids (without CFO–BTO incubation). Further SEM analysis verified that the morphological integrity of the NPCs was preserved following incubation with CFO–BTO (Fig. 2i). These findings collectively suggest that CFO–BTO nanoparticles are capable of adhering to NPC membrane surfaces without disrupting the cellular architecture, thereby indicating their potential to enhance further differentiation capabilities. Additionally, NPCbots exhibited an increased presence of filopodia (Supplementary Fig. 6), probably resulting from mild interactions between the particles and cell membranes. These interactions are beneficial for NPCbot migration, environmental sensing and adherence to the extracellular matrix^38^.

## AMF drives neuronal differentiation of NPCbots

**Fig. 3:**
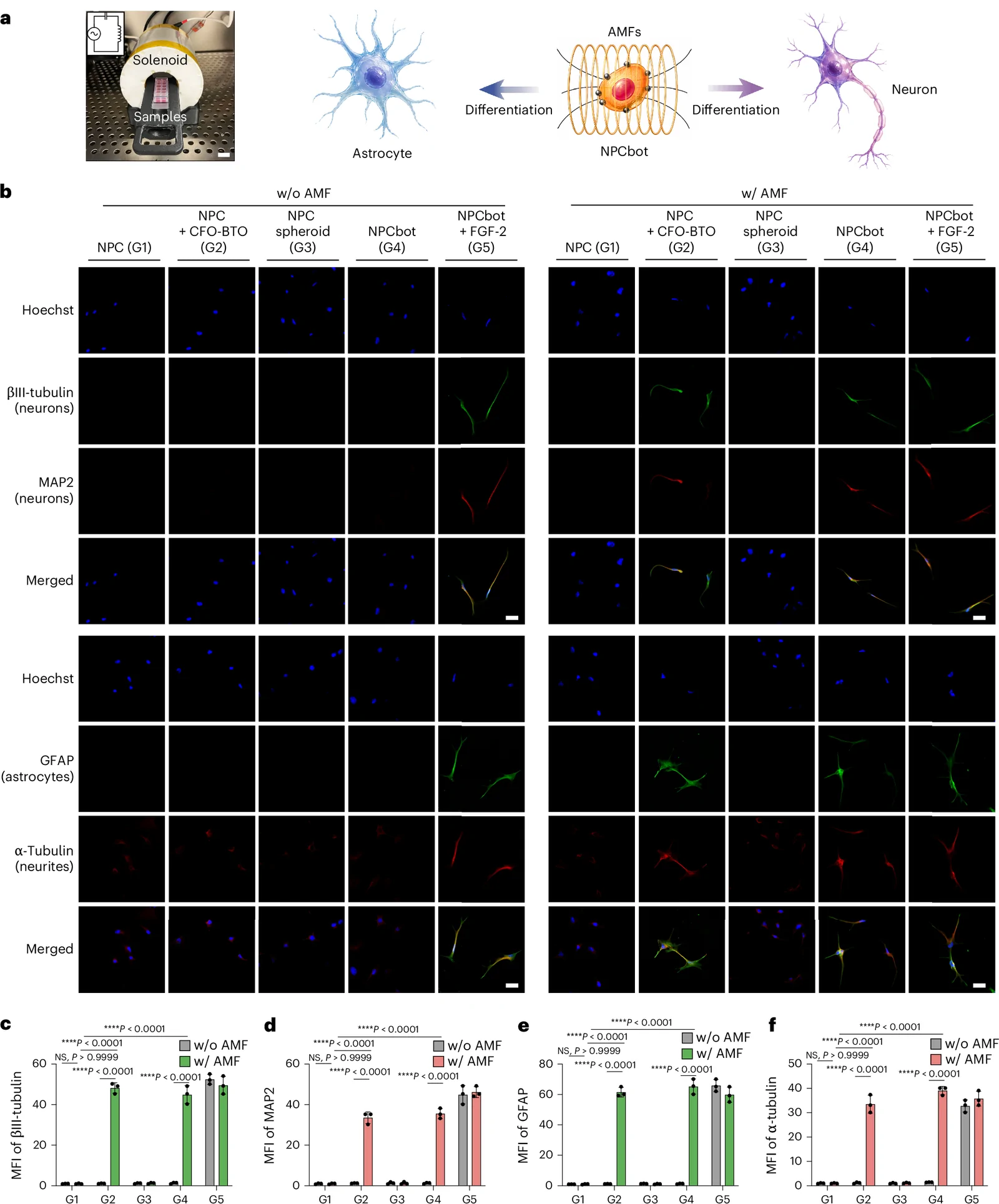
Induction of NPCbot differentiation using AMF. **a**, Schematic of the experimental setup for differentiation induction. NPCbots were exposed to AMFs generated by a solenoid coil with a 5-cm internal diameter. Scale bar, 20 mm. The coil operated within a resonant tank circuit, producing a magnetic field with a peak amplitude of 20 mT and a frequency of 1.18 kHz at the centre of the solenoid. **b**, Immunofluorescence analysis of NPCbots showing the expression of neuronal markers. The samples were stained for βIII-tubulin and MAP2 to identify differentiated neurons and stained for α-tubulin and GFAP to identify differentiated neurites and astrocytes, respectively. Scale bars, 8 μm. **c**–**f**, Quantitative assessment through mean fluorescence intensity (MFI) across all samples (*n* = 3 experiments; data are presented as mean ± s.d.). All MFI values were normalized to the G1 w/o AMF control. Statistical significance was calculated via one-way analysis of variance (ANOVA) with a Tukey’s post hoc test. w/o, without; w/, with; NS, not significant. Source data

To investigate the differentiation potential of NPCbots under specific conditions, we used an AMF stimulation protocol. Our experimental setup involved placing samples within a solenoid coil with an internal diameter of 5 cm, operating in a resonant tank configuration (Fig. 3a). This configuration generated a magnetic field with an amplitude of 20 mT and a frequency of 1.18 kHz at the centre of the coil. The AMF was applied for 2 h, twice daily, for 2 days to realize the NPCbot differentiation. We used fibroblast growth factor 2 (FGF-2), a neuronal differentiation inducer^39^, as a positive control in these experiments. Immunofluorescence imaging conducted post-AMF treatment revealed a shift of NPCbots towards neuronal cell types. Figure 3b–d showcases these transformations, accompanied by an enhanced expression of neuronal markers, βIII-tubulin and microtubule-associated protein 2 (MAP2). Quantitative analysis indicated a significant increase in the expression of both markers, with the mean fluorescence intensity rising by 35.3-fold and 28.0-fold for βIII-tubulin and MAP2, respectively, compared with controls without AMF treatment. These markers are integral to neuronal cell structure and function^40,41^, signifying effective neural induction through AMF stimulation. Figure 3e,f shows that NPCbots also demonstrated a significant upregulation of α-tubulin and glial fibrillary acidic protein (GFAP) post-treatment, with mean fluorescence intensity increases of 27.5-fold and 49.8-fold, respectively. α-Tubulin is indicative of neurite outgrowth, whereas GFAP is a marker for astrocytes^40,42^, indicating that the NPCbots not only differentiate into neurons but also promote astrocytic lineage development under the influence of AMF. Additionally, significant improvements were observed in the NPC + CFO–BTO group treated with AMF, mirroring these enhancements. These results elucidate that the magnetoelectric properties of the CFO–BTO nanoparticles, situated on the NPC membrane within the NPCbots, respond to an AMF by generating electric charges, which probably influence calcium channel activity and trigger calcium influx^23^. This influx is presumed to initiate signalling pathways that foster the following differentiation^43^, underscoring the potential of AMF to regulate cellular behaviour, which provides a non-invasive method to direct cellular fate. Additional mechanistic evidence supporting AMF-induced NPCbot differentiation is provided in Supplementary Note 1 and Supplementary Figs. 7–9. Longer-term in vitro analyses showed sustained NPCbot viability over 14 days, together with progressive neuronal differentiation at 7 days and functional maturation at 14 days under prolonged AMF stimulation (Supplementary Note 2 and Supplementary Figs. 10–12).

## NPCbots support zebrafish spinal cord repair

**Fig. 4:**
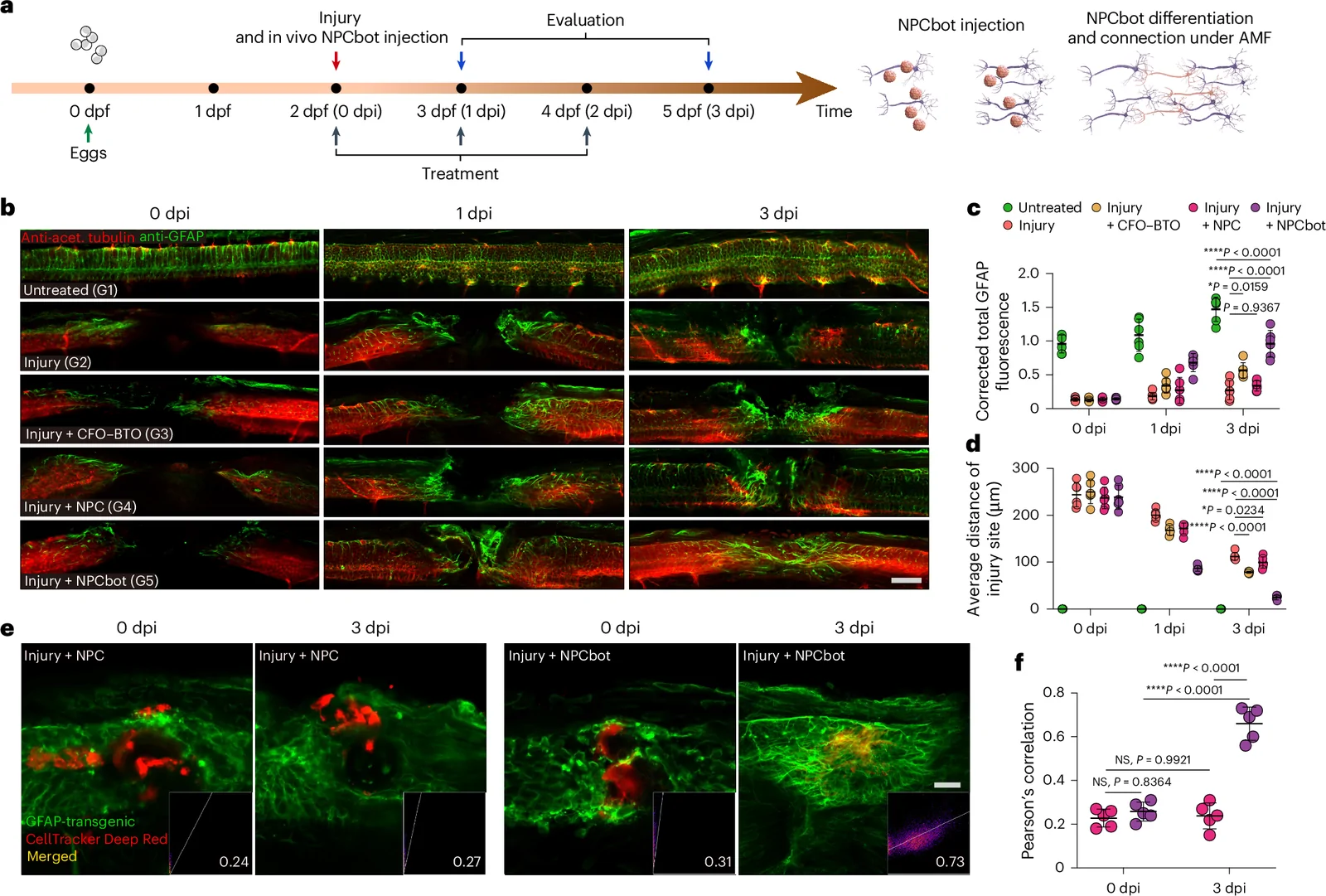
Therapeutic efficacy of NPCbots in treating zebrafish SCIs. **a**, Schematic of the experimental design for treatment processes. **b**, Representative whole-mount immunohistochemistry images demonstrating the co-labelling of neurites (anti-acetylated tubulin, red) and the GFAP (green) at the site of injury, assessed at 0, 1 and 3 dpi following different treatments under AMF. Scale bar, 50 μm. **c**, Quantitative analysis of the corrected total intensity of GFAP in various experimental groups at 0, 1 and 3 dpi (*n* = 6 per group). **d**, Mean distance from the injury site observed in different treatment groups at 0, 1 and 3 dpi. For each fish, measurements were taken at five equidistant points from the top to the bottom of the injury site, and the average of these measurements was calculated (*n* = 6 per group). **e**, Live imaging of the colocalization of GFAP in a transgenic line with NPC/NPCbot labelled with CellTracker Deep Red and the corresponding scatter plot with Pearson’s correlation coefficient. Scale bar, 40 μm. **f**, Quantitative evaluation of colocalization, using Pearson’s correlation coefficient (*n* = 5 per group). Statistical significance was calculated via one-way ANOVA with a Tukey’s post hoc test (**c**, **d** and **f**). Data are presented as mean ± s.d. Source data

To assess the therapeutic efficacy of NPCbot treatment, we established a zebrafish SCI model^44^. This model was utilized to evaluate the neural repair potential of NPCbots administered via injection and subsequently stimulated with AMF during a 5-day experimental window. Although zebrafish larvae possess innate spinal cord regeneration capabilities, our modelling strategy demonstrated that by the endpoint of the experiment at 5 days post-fertilization (dpf), the spinal cord had not fully recovered (Supplementary Fig. 13). As depicted in Fig. 4a, the experimental timeline was designed to allow for a comprehensive assessment of recovery following injury at 2 dpf. Stimulation was conducted for 2 h each day from 2 to 4 dpf. The magnetic field direction was oriented parallel to the longitudinal axis of the zebrafish. Using whole-mount immunohistochemistry, we documented the therapeutic benefits in zebrafish treated with NPCbots (Fig. 4b). In particular, the group treated with CFO–BTO nanoparticles (G3) exhibited enhanced fluorescence intensity of GFAP at the injury site (Fig. 4c), indicative of an active astrocytic response that may support axonal regrowth^45^. This was coupled with a noticeable reduction in lesion distance (Fig. 4d), which may be attributed to the electric charges generated by CFO–BTO under the influence of AMF, which, in turn, facilitated neural repair. Correspondingly, these findings are consistent with the NPC differentiation results from our in vitro cell stimulation test. A marked recovery was most pronounced in the NPCbot-treated group (G5). α-Tubulin staining demonstrated a robust reformation of neuronal structures, crucial for maintaining neural function and integrity (Supplementary Fig. 14a,b). Concurrently, α-tubulin and GFAP staining of the NPCbot-treated group (G5) highlighted a significant presence of neural connections compared with the control groups (injured (G2), CFO–BTO treated (G3) and NPC treated (G4)), showcasing the greatest rate of change from 1 day post-injury (dpi) to 3 dpi in corrected total GFAP and α-tubulin fluorescence, as well as the smallest average distance at the injury site (Supplementary Fig. 14c–e). This underscores that combining the NPCbot strategy is crucial in aiding the zebrafish’s spinal cord to repair itself (Fig. 4b–d).

Further supporting a role for NPCbots in astrocyte engagement during neural repair, we tracked how effectively our NPCbot treatment integrated into this spinal repair mechanism. Additional analyses involved live imaging in conjunction with a zebrafish transgenic line labelling GFAP to monitor astrocyte dynamics. We tracked the progression of regeneration with and without NPCbot treatment in the same fish, exploring the comparative impact of our intervention (Fig. 4e). Comparing the NPCbot group with the control group treated with NPCs only, we observed a 2.6-fold increase in the colocalization of GFAP expression and NPC/NPCbot cells labelled with CellTracker Deep Red by 3 dpi. This colocalization, evident in the merged yellow fluorescence in the images, underscored an enhanced integration of differentiated NPCbots with host astrocytes. The Pearson’s correlation coefficient, a measure of colocalization, indicated a 2-fold increase at 3 dpi in the NPCbot-treated group compared with the controls (Fig. 4f). This finding confirms that NPCbots not only differentiate into astrocytic lineages expressing GFAP but also actively incorporate into the healing spinal tissue, thereby potentially facilitating more effective repair under AMF stimulation.

## NPCbots promote zebrafish motor recovery

In this segment of our study, we carried out extensive behavioural assessments to ascertain both structural and functional recovery in zebrafish following SCI and subsequent treatment with NPCbots. Utilizing a 96-well plate, we divided the wells to accommodate six zebrafish groups subjected to different treatment protocols, with each group allocated 16 wells. Observations spanned a 15-min period, segmented into alternating light and dark cycles of 5 min each, to gauge zebrafish responses to environmental light shifts (visual motor response), a vital parameter for assessing the efficacy of NPCbot interventions on zebrafish behaviour. We measured swimming distance and speed, both average and peak, alongside the movement patterns.

**Fig. 5:**
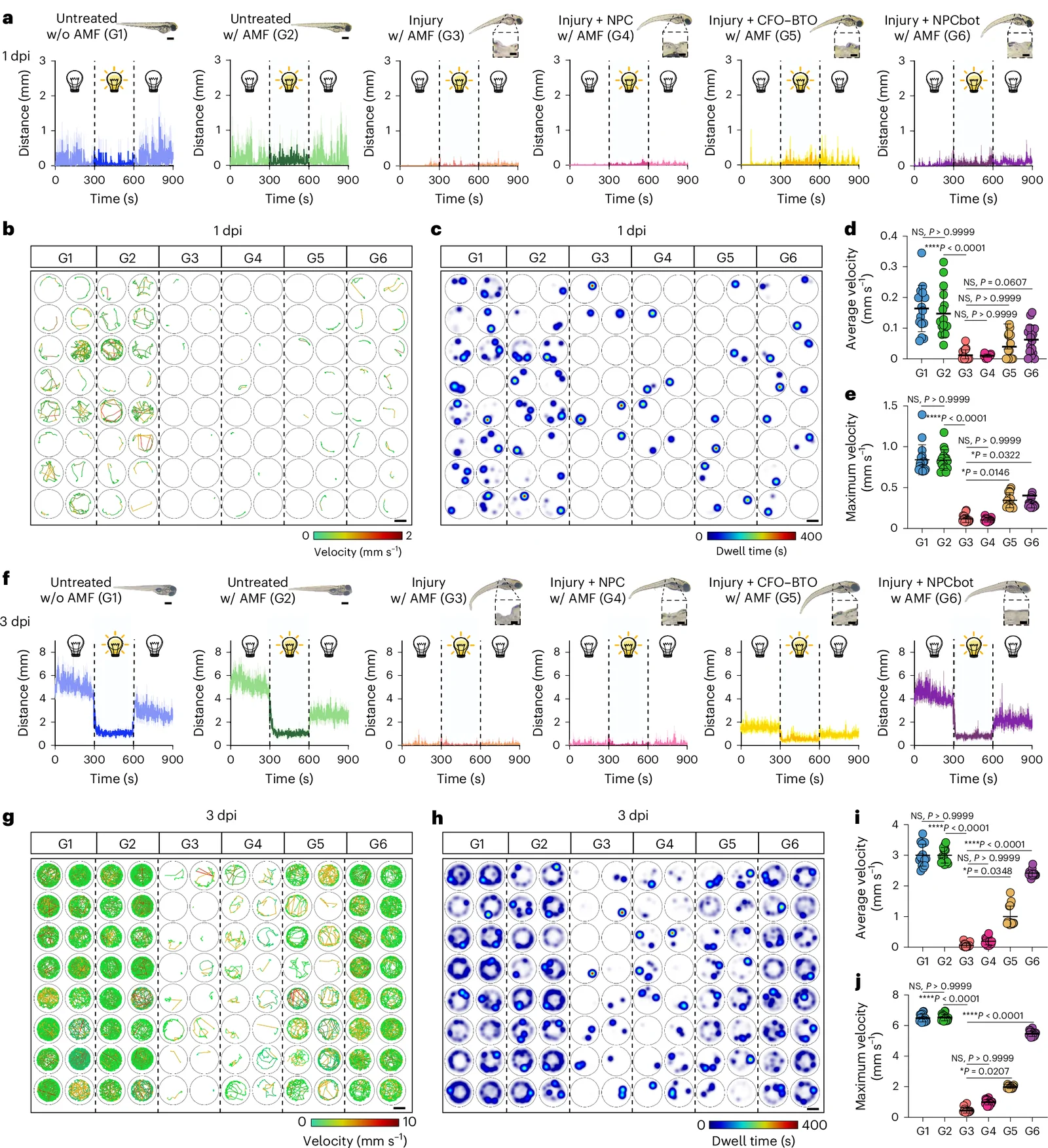
Behavioural assessment in NPCbot-treated zebrafish larvae. **a**, Representative images of zebrafish larvae at 1 dpi after different treatments, with swimming distance assessed using a dark–light–dark protocol, each phase lasting 5 min. Scale bars, 1 mm. *n* = 16 per group; data are presented as mean ± s.e.m. **b**,**c**, Trajectory (**b**) and heat map (**c**) illustrating the movement patterns of differently treated larvae. Scale bars, 4 mm. **d**,**e**, Quantitative analysis of the average (**d**) and maximum swimming speeds of the treated larvae (**e**). *n* = 16 per group; data are presented as mean ± s.d. **f**, Representative images of zebrafish larvae at 3 dpi after different treatments, with swimming distance assessed using the same dark–light–dark protocol. Scale bars, 1 mm. *n* = 16 per group; data are presented as mean ± s.e.m. **g**,**h**, Trajectory (**g**) and heat map (**h**) illustrating the movement patterns of differently treated larvae. Scale bars, 4 mm. **i**,**j**, Quantitative analysis of the average (**i**) and maximum swimming speeds of the treated larvae (**j**). *n* = 16 per group; data are presented as mean ± s.d. Statistical significance was calculated via one-way ANOVA with a Tukey’s post hoc test (**d**, **e**, **i** and **j**). For the trajectory analysis (**b** and **g**), red indicates an increased swimming speed, whereas green denotes a decreased speed. In the heat-map analysis (**c** and **h**), red signifies increased dwell time, and blue signifies decreased dwell time. Source data

At 1 dpi, there was a notable disparity between the healthy (G1, G2) and injured groups (G3–G6). Unlike the healthy groups, the injured groups exhibited significantly reduced movement or paralysis in more severe cases (Fig. 5a–e). This diminished mobility underscores the critical role of the spinal cord in relaying movement signals from the brain to the body. Moreover, injured fish demonstrated general impairments in swimming behaviour under varying light conditions and did not show noticeable motion, as indicated by their trajectories in Fig. 5b. Heat-map analysis further delineated these behavioural modifications, accentuating areas within the wells in which zebrafish lingered most frequently (Fig. 5c). By day 3 (1 dpi), healthy zebrafish displayed increased activity compared with the other groups, with movement patterns that suggest broader mobility within their designated wells. Conversely, those suffering from spinal injuries displayed a stark contrast, predominantly remaining immobile and confined to limited areas, indicative of their compromised motor functions and altered exploratory behaviour.

Significant enhancement was evident by day 5 (3 dpi) in the NPCbot-treated group (G6), compared with those receiving only nanoparticle treatment (G5) or left untreated (G3). NPCbot-treated zebrafish not only demonstrated elevated overall swimming speeds and distances but also displayed movement difference in response to different light conditions, indicative of a resurgence in neural function (Fig. 5f–j). The behavioural data extracted from Zebrabox, an automated observation chamber and behavioural tracking system for zebrafish, unequivocally showed marked improvements in motor function, with not only facilitated structural repair but also restored functional behaviours. Further analysis of swimming patterns showed a tendency among the NPCbot-treated zebrafish to swim along the tank edges in circles, a behaviour suggesting enhanced curiosity and improved spatial awareness (Fig. 5h). This behaviour not only mirrors better motor function but also hints at a recovery in sensory and neural processing capabilities, facilitating more effective interaction with their surroundings. Additionally, it is noteworthy that NPC treatment alone (G4) did not significantly enhance the recovery of motor behaviours in injured zebrafish.

Together, these detailed behavioural assessments and quantitative analyses substantiate that NPCbot treatment significantly aids in the recuperation of motor functions and behavioural patterns in zebrafish post-SCI. The reinstatement of almost normal swimming behaviour and improved exploratory behaviours in NPCbot-treated zebrafish underscore the potential of this innovative treatment strategy in fostering comprehensive neural repair. These findings not only emphasize the efficacy of NPCbots in structural neural repair but also highlight their role in the functional restoration of the central nervous system.

## NPCbots improve repair and motor function in mice

**Fig. 6:**
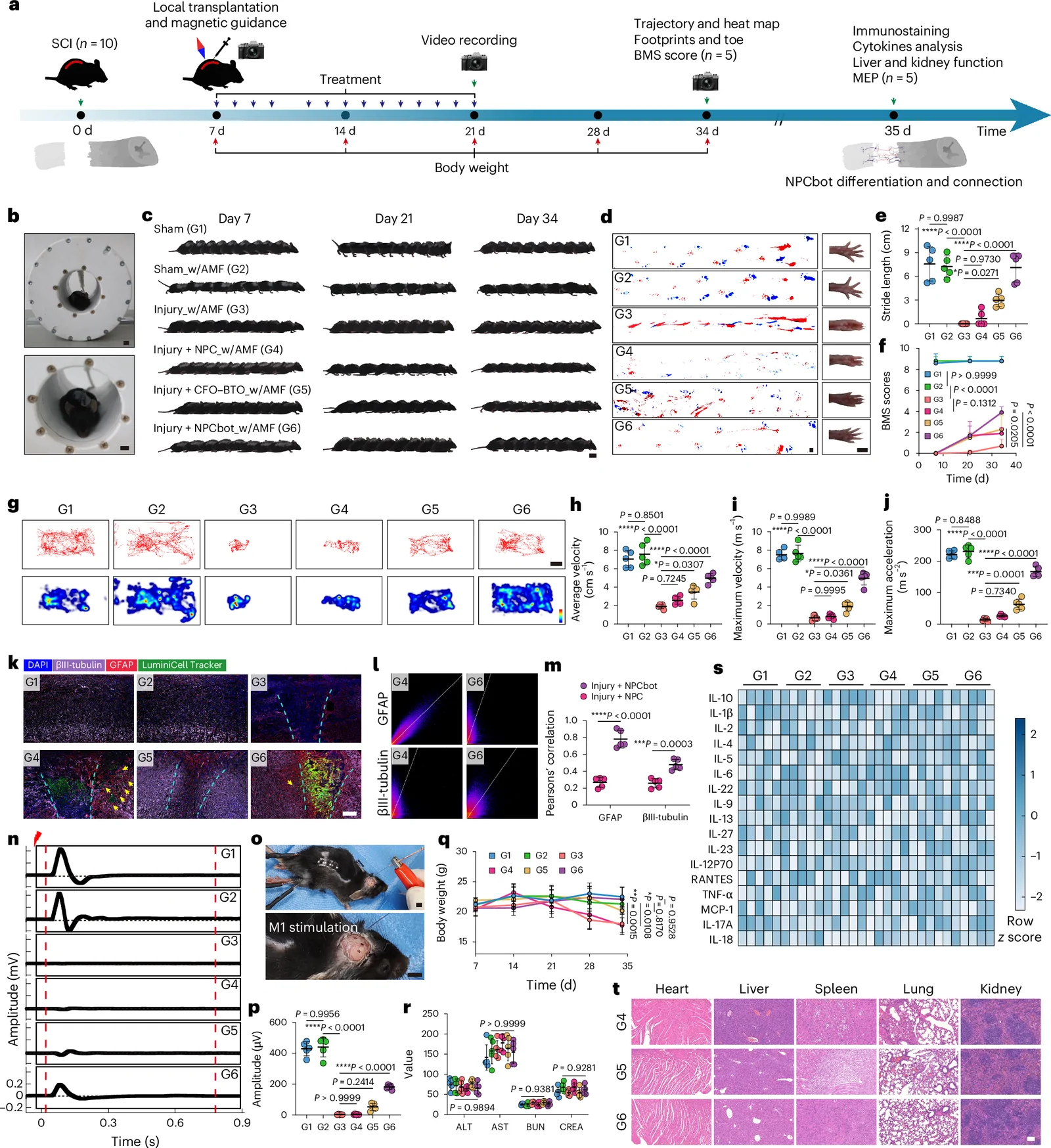
Therapeutic efficacy of NPCbots in treating murine SCI. **a**, Schematic of the treatment design. **b**, Magnetic stimulation setup. Scale bars, 1 cm. **c**, Representative photographs of treated mice. Scale bar, 3 cm. **d**, Representative footprints and toe spreading on day 34, with forelimbs in blue and hindlimbs in red (*n* = 5). Scale bars, 7 mm. **e**, Stride length, measured as the distance between two successive placements of the same hindlimb (*n* = 5 per group). **f**, BMS scores for hindlimb function over time (*n* = 5 per group). **g**, Representative trajectories and heat maps in the behaviour observation box on day 34 (*n* = 5). Scale bar, 5 cm. **h**–**j**, Average speed (**h**), maximum speed (**i**) and maximum acceleration (**j**) on day 34 (*n* = 5 per group). **k**, Immunofluorescence analysis of the spinal cord on day 35. Scale bar, 400 μm. Lesion sites are outlined by cyan dashed lines, and transplanted NPCs or NPCbots are marked by yellow arrows. **l**, Colocalization of LuminiCell Tracker-labelled NPCs or NPCbots with neurons (βIII-tubulin) and astrocytes (GFAP). **m**, Quantification of colocalization by Pearson’s correlation coefficient (*n* = 5 per group). **n**,**o**, MEP values recorded from the gastrocnemius on day 35 after M1 stimulation with a 0.5 mA, 0.5 ms square wave. Scale bars, 5 mm. **p**, MEP peak amplitude (*n* = 5 per group). **q**, Body weight over time (*n* = 5 per group). **r**, Hepatorenal function parameters on day 35 (*n* = 5 per group). **s**, Luminex-based quantifications of cytokines and chemokines on day 35 (*n* = 5 per group). **t**, Haematoxylin and eosin staining for major organs on day 35 (*n* = 5 per group). Scale bar, 50 μm. Statistical significance was calculated via one-way ANOVA with a Tukey’s post hoc test. Data are presented as mean ± s.d. Source data

We subsequently expanded our research to include non-regenerating mammalian species to evaluate the repair potential of NPCbots. Figure 6a illustrates the experimental protocol used in C57BL/6 mice to establish a model of SCI. Initially, a laminectomy was performed at the T10 vertebra to constitute the sham group. This procedure was followed by the complete excision of a 2-mm spinal cord segment at the T10 vertebral level, thereby creating the injury group. Following a recovery period of 7 days, therapeutic agents were administered directly at the site of injury. Treatment involved the localized injection of a solution containing 5 × 10^5^ NPCbots suspended in 5 μl of fibrin gel. Concurrently, magnetic guidance was applied at the injury site to achieve the microscale intralesional positioning of NPCbots. Subsequently, alternating magnetic stimulation was administered for 30 min daily from days 7 to 21. The magnetic field was oriented parallel to the longitudinal axis of the spinal cord (Fig. 6b).

To assess functional recovery post-injury (Fig. 6c and Supplementary Video 1), all groups, except for the sham group, initially presented with a pronounced limp on day 7 post-injury. Following 2 weeks of magnetic stimulation, the NPCbot-treated group began exhibiting signs of locomotor improvement by day 21. By day 34, both CFO–BTO and NPCbots groups showed marked improvements in their walking gaits, with the NPCbots group’s recovery being more pronounced. The toe spreading score was evaluated on day 34. The group treated with NPCbots (G6) demonstrated improved toe spreading function, achieving almost complete abduction, whereas the other treatment groups displayed varying degrees of contracture (Fig. 6d and Supplementary Fig. 15). Subsequent behavioural assessments involved footprint analysis, which revealed that by day 34, mice treated with CFO–BTO (G5) exhibited increased stride lengths. In particular, mice in the NPCbots group (G6) not only displayed significant enhancements in walking patterns but also achieved the longest strides among all treatment groups (Fig. 6e). The Basso mouse scale (BMS) was utilized to quantify hindlimb motor function in mice following SCI (Fig. 6f). The CFO–BTO-treated mice (G5) showed a substantial increase in average BMS scores, registering at 2.3, compared with scores of 0.7 and 1.9 for the untreated (G3) and NPC-treated mice (G4), respectively. The NPCbot-treated mice (G6) exhibited the most effective repair of SCI, achieving an average BMS score of 3.9, in contrast to the sham (G1 and G2) score of 8.8. This demonstrates that NPCbot treatment resulted in the most effective recovery of overall mobility post-surgery.

Further behavioural analysis involved an 8-min observation period in an exploratory box to assess the exploratory behaviour and general activity levels of the mice from each group (Fig. 6g). The NPCbot-treated group (G6) was observed actively exploring their environment and exhibited the longest walking trajectories compared with other treated groups. Conversely, groups receiving other treatments showed restricted movement, confined to smaller areas. The analysis of trajectory and heat-map data allowed for the quantification of speed and acceleration improvements, confirming that the NPCbot-treated group (G6) showed the most significant enhancements in these parameters compared with the groups with untreated injuries (Fig. 6h–j).

Following behavioural assessments, mice were euthanized to collect spinal cord specimens for histological examination to elucidate the therapeutic impacts and underlying molecular mechanisms. Figure 6k illustrates that untreated mice (G3) exhibited no detectable neurite bridging across the lesion. By contrast, mice treated with CFO–BTO (G5) displayed edge-biased neurite ingrowth, with βIII-tubulin-positive processes extending shallowly from the stumps into the gel rim without spanning the cavity, presumably because the progressive fibrin remodelling concentrating CFO–BTO at the tissue–gel interface, where AMF-driven, charge-mediated stimulation may promote periphery-to-centre neurite extension. In particular, the NPC group (G4) showed limited in vivo differentiation and a dispersed cell distribution in the absence of magnetic guidance and stimulation. However, the NPCbot-treated mice (G6) demonstrated pronounced localization at the injury site, exhibiting marked neuronal and astrocyte-like differentiation and enhanced local connectivity, as evidenced by colocalization with neuronal and astrocyte markers. Quantitative analysis using Pearson’s correlation coefficient based on immunofluorescence results revealed significantly increased colocalization with neurons and astrocytes following NPCbot treatment (Fig. 6l,m). These findings underscore the superior potential of NPCbots to effectively repair the injury site.

To further elucidate the mechanisms underlying neural activities, we evaluated the impact of NPCbots on the functional integrity of the corticomotor system by conducting motor-evoked potential (MEP) testing (Fig. 6n,o). For this purpose, a stimulation electrode was precisely positioned within the sensorimotor cortex, and responses were monitored using electrodes placed on the gastrocnemius muscle and tail. The groups that sustained injuries, along with those treated with CFO–BTO (G5) or NPC (G4), exhibited significantly weaker MEP signals compared with the sham groups (G1 and G2). This reduction was characterized by markedly reduced MEP peak amplitudes. In particular, the NPCbot group (G6) demonstrated enhanced MEP signals, with a significant increase in amplitude (Fig. 6p), indicating improved corticomotor responsiveness and superior recovery outcomes.

For the biosafety of the NPCbots, we evaluated the body weight dynamics and observed a notable decrease in the body weight of both untreated mice (G3) and NPC-treated mice (G4) by day 35 (Fig. 6q). Conversely, the mice treated with CFO–BTO (G5) and NPCbot (G6) exhibited stable body weights throughout the observation period. Furthermore, we assessed liver and kidney functions by testing four key indicators. The results revealed no significant alterations in these parameters across all experimental groups (Fig. 6r), indicating that liver and kidney functionalities were preserved. Cytokine levels in serum were quantified using Luminex-based assays to investigate potential immunogenic responses (Fig. 6s). Our findings indicated that cytokines and chemokines levels remained consistent across all treatment groups, suggesting that the treatments did not elicit notable immune reactions. The biocompatibility of the NPCbots was further substantiated through the histological analyses of five major organs. Haematoxylin and eosin staining demonstrated that none of the treatments inflicted damage on the major organs (Fig. 6t and Supplementary Fig. 16), confirming the safety and biocompatibility of our approach in the mouse model over 4 weeks.

## NPCbot can be magnetically guided in vitro and in vivo

**Extended Data Fig. 1:**
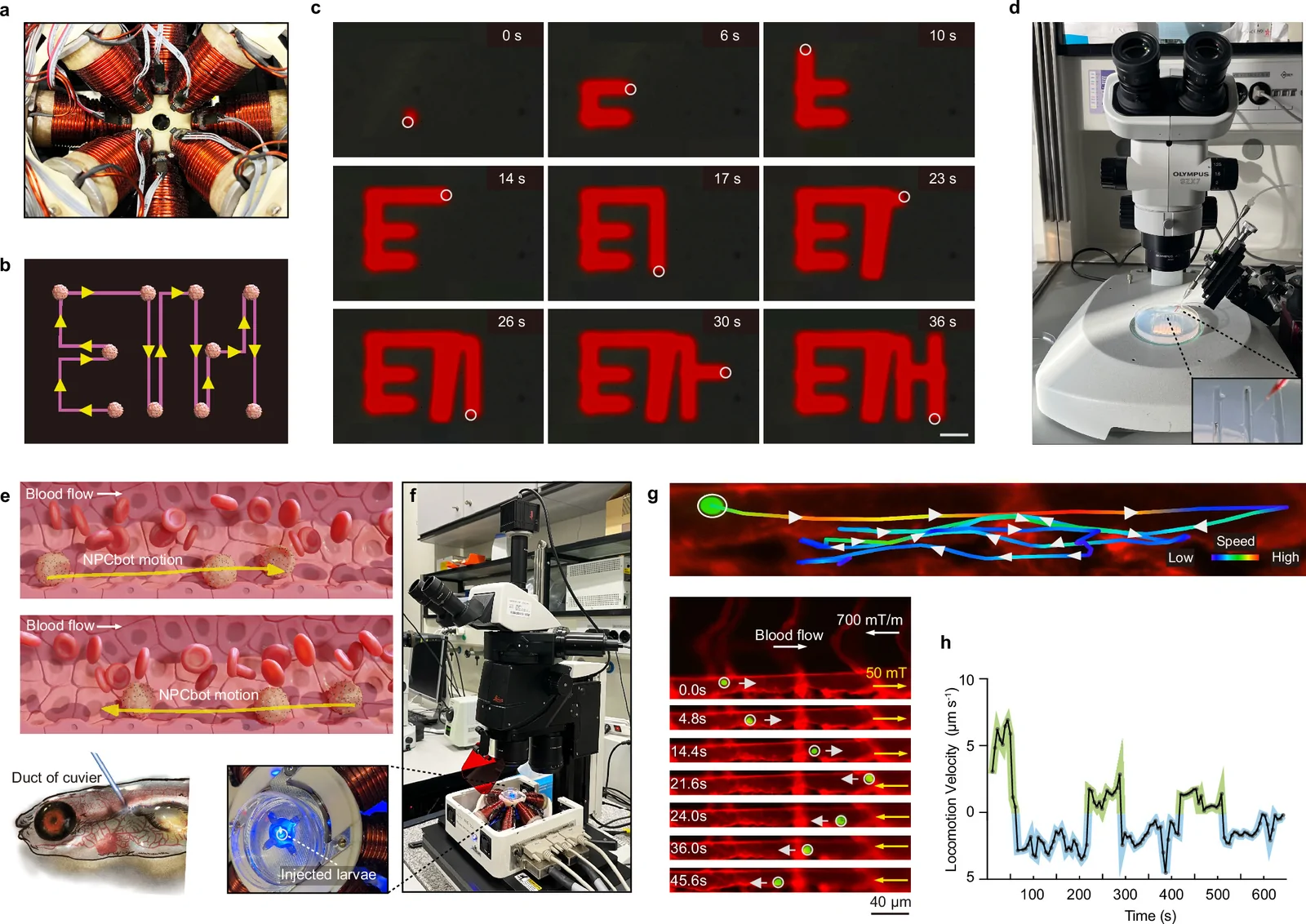
*In vitro* and *in vivo* manipulation of NPCbots. **a**, Photograph of the magnetic manipulation apparatus, which enables five degrees of freedom (5-DOF) for wireless micromanipulation. **b**, Preset motion trajectory for the NPCbot. **c**, Microscope images illustrating the locomotion of magnetic rolling NPCbots under an external rotating magnetic field of 10 mT at 1 Hz in a phosphate-buffered saline (PBS) solution, with the NPCbots’ positions highlighted by white circles. Scale bar, 20 μm. **d**, Photographs of the injection setup. **e,f**, Schematic of the *in vivo* manipulation setup and navigation scenario following injection into zebrafish larvae, showing how the NPCbot can navigate both with and against the blood flow. **g**, Trajectory analysis of NPCbot movement within the dorsal aorta (DA) of kdrl:mCherry zebrafish larvae, highlighted by white arrows indicating the direction of travel. The NPCbots, labeled with live dye in green, are depicted navigating both with and against the blood flow. This was achieved by applying a rotating magnetic field of 50 mT and a compensatory magnetic field gradient of 700 mT/m to counteract the hemodynamic forces. The stereomicroscope images (below) capture the real-time positioning of the NPCbots within the fluorescently highlighted vascular architecture. Scale bar, 40 μm. **h**, Analysis of NPCbot locomotion velocity within the DA of zebrafish larvae, as measured using Imaris software. The green-shaded regions represent the NPCbot movement in the same direction as the blood flow (co-directional navigation), and the blue-shaded regions represent movement opposite to the blood flow (counter-directional navigation). Source data

To evaluate the translational potential of NPCbots for clinical applications, we explored their navigation capabilities both in vitro and in vivo. Utilizing a state-of-the-art magnetic manipulation system (MFG-100, MagnebotiX AG) capable of five-degrees-of-freedom wireless micromanipulation (Extended Data Fig. 1a), we assessed the motility of NPCbots within a phosphate-buffered saline (PBS) solution. The results (Extended Data Fig. 1b,c) and further illustrated in a video (Supplementary Video 2) demonstrate that under a rotating magnetic field of 10 mT at a frequency of 1 Hz, NPCbots can execute a rolling motion to follow a predefined trajectory, spelling ‘ETH’ in this instance, within a glass Petri dish. Supplementary Video 3 additionally demonstrates the controlled movement of multiple NPCbots. The NPCbots displayed reliable magnetic responsiveness, navigating the set path with minimal deviation, which highlights their potential for precise path-following capabilities in fluidic environments.

Transitioning to in vivo experiments, NPCbots were prelabelled using a live dye (L3224, LIVE/DEAD Viability/Cytotoxicity Kit, Thermo Fisher Scientific) and introduced into the duct of Cuvier in zebrafish embryos at 2 dpf by injection of 2–3 nl of NPCbot solution using a FemtoJet injection system (Eppendorf) (Extended Data Fig. 1d). A rotating magnetic field of 50 mT at 3 Hz, coupled with a compensatory magnetic field gradient of 700 mT m^−1^, was used to counteract the influence of blood flow within the dorsal aorta (DA), a major vessel in zebrafish embryos characterized by high flow rates. This setup allowed for the precise manipulation of NPCbots within the vascular system. The in vivo locomotion of the NPCbots was meticulously documented utilizing a Leica M205 FCA stereomicroscope (Extended Data Fig. 1e,f). To further scrutinize the movement dynamics of the NPCbots in vivo, we used the kdrl:mCherry transgenic line to visualize blood vessels, whereas the NPCbots were distinctly labelled with live dye (FITC channel). Our comprehensive analysis (Extended Data Fig. 1g) demonstrates that the NPCbots could be manipulated within the designated area. The NPCbots performed controlled back-and-forth movements (Supplementary Video 4), spanning a distance of 112 µm. Despite the dynamic environment posed by the blood flow within the DA, the NPCbots travelled a maximum distance of 156 μm from their initial position, indicating effective navigational control.

Furthermore, the locomotion of NPCbots within the DA was systematically analysed over a 600-s interval (Extended Data Fig. 1h). When moving with the blood flow (green curve), NPCbots occasionally reached peak speeds of nearly 8 µm s^−1^, probably benefiting from the additive velocity of the blood flow. However, these peak speeds were not consistently maintained. Conversely, the movement against the blood flow (blue curve) showcased generally lower speeds but greater stability. This pattern of movement indicates a consistent resistance from the opposing blood flow, which limits peak speeds but results in a steadier navigation pattern.

Additionally, Supplementary Video 5 displays controlled movement in the pericardium of multiple NPCbots. The navigation analysis underscores the capabilities of NPCbots for targeted navigation and actuation within both in vitro and in vivo environments. The precise control achieved over NPCbot movement, especially within complex biological systems like the zebrafish vascular network, highlights the potential of magnetic microrobots in therapeutic applications, including targeted drug delivery and regenerative medicine.

## Outlook

The development and application of NPCbots described in this study introduce a platform for preclinical SCI models. By integrating microfluidics technology with magnetoelectric nanoparticles and using real-time magnetic navigation, this approach may provide a method for precision therapeutic delivery and cellular manipulation. This approach may help transform treatment paradigms not only for SCIs but also for a broader spectrum of neurodegenerative diseases. With further validation in larger animal models, NPCbots could play a valuable role in the treatment of neurological disorders and injuries by enabling magnetically guided, efficient and minimally invasive interventions. Moreover, the conceptual and technological frameworks developed in this study may inspire similar approaches in other medical fields. The combination of targeted delivery and controlled therapeutic action demonstrated here may also inform future strategies in areas such as oncology and cardiology, where precise localization and controlled intervention are crucial. Further translation of the NPCbot platform will require scalable production, long-term safety evaluation and continued advances in imaging and real-time tracking.

## Methods

All procedures align with ethical regulations. All zebrafish experiments were conducted in accordance with the regulations of the Veterinary Authorities of Kanton Zurich (TV4206). Male C57BL/6 mice were housed at the Animal Center of Shenyang Pharmaceutical University. All mice experimental procedures were conducted in strict compliance with the guidelines approved by the Institutional Animal Ethical Care Committee (IAEC) of the university (SYXK 2021-0009).

### Synthesis of CFO–BTO core–shell magnetoelectric nanoparticles

CFO–BTO core–shell nanoparticles were synthesized through a sequential application of co-precipitation, hydrothermal and sol–gel methods, as detailed in prior studies^33^. Initially, 2 g of cetyltrimethylammonium bromide was dissolved in 30 ml of deionized water. Following this, 1 g of ferric chloride hexahydrate (FeCl_3_·6H_2_O) and 0.24 g of anhydrous cobalt(II) chloride (CoCl_2_) were separately dissolved in the cetyltrimethylammonium bromide solution. A 6-M sodium hydroxide (NaOH) solution was then gradually added to initiate the precipitation of CFO nanoparticles. For the transformation of the CFO nanoparticles into a single-crystalline structure, the resultant mixture was sealed in an autoclave and subjected to a hydrothermal treatment at elevated temperatures. Subsequently, the formation of the BTO shell over the CFO core was achieved using a sol–gel process. The BTO precursor was prepared by combining 30 ml of a deionized water solution containing 0.029 g of barium carbonate and 0.1 g of citric acid with 30 ml of an ethanolic solution, which included 1 g of citric acid and 0.048 ml of titanium isopropoxide. This mixture was continuously stirred at 90 °C for 1 h to ensure complete sol formation. CFO nanoparticles, previously dispersed via sonication for 2 h, were added to the sol to form a gel. This gel was dried at 80 °C overnight. The dried gel was subsequently annealed at 600 °C to yield CFO–BTO nanoparticles.

### Characterization of CFO–BTO core–shell magnetoelectric nanoparticles

The morphology of the nanoparticles was examined using a scanning transmission electron microscope (FEI Talos F200X). The elemental distribution within the nanoparticles was assessed by HAADF-STEM on a FEI Talos F200X system with EDX spectroscopy for elemental mapping. X-ray diffraction analyses were performed using an Empyrean diffractometer, which utilizes a copper X-ray source (*λ* = 1.5406 Å) and is coupled with a PIXcel detector (Malvern Panalytical). Local piezoresponse loops were evaluated using piezoresponse force microscopy (NT-MDT Ntegra Prima). A conductive atomic force microscopy tip (FMG01/Pt) in the contact mode applied an alternating voltage of 0.5 V to the CFO–BTO NP, inducing piezoelectric surface oscillations. These oscillations were detected via cantilever deflection. Local piezoresponse hysteresis loops were generated at random nanoparticle locations by modulating the applied d.c. bias and simultaneously recording the phase and amplitude responses. The excitation voltage was a stepwise increasing pulsed d.c. voltage, augmented with a small alternating-current (a.c.) voltage to mitigate electrostatic interference, with the a.c. response signal captured exclusively during the off phase of the pulse sequence. To investigate changes in the piezoelectric response under magnetic influence, an in-plane magnetic field of 50 mT was applied to the sample.

### Cell culture

In the protocol for cultivating iPSC-derived NPCs, the utilization of Matrigel-coated culture vessels was crucial. The process commenced with the careful thawing of Matrigel at reduced temperatures, followed by a dilution at a ratio of 1:50 with chilled Dulbecco’s modified Eagle’s medium. This mixture was subsequently applied to culture flasks, which were then incubated overnight within a temperature range of 4 °C. Before cell seeding, the coating solution was aspirated and replaced with prewarmed ENStem-A Neural Expansion Medium (catalogue number SCM004a, Merck). Cryopreserved human iPSC-derived NPCs (catalogue number SCC035, Merck) were swiftly thawed at 37 °C in a water bath and then transferred into a sterile 15-ml conical tube. Here they were gently mixed with prewarmed medium to mitigate osmotic shock. The cell suspension was subsequently centrifuged, the supernatant was decanted and the cell pellet was resuspended. Cell viability was assessed through counting before seeding onto the prepared Matrigel-coated culture vessels. Cultures were maintained at 37 °C in a humidified atmosphere containing 5% CO_2_, with medium exchanges every 2 days. On attaining confluence, NPCs were dissociated using Accutase (catalogue number SCR005, Merck), quantified and replated into similarly prepared T75 flasks. This process promoted optimal conditions for the proliferation and maintenance of NPCs. Stemness was authenticated by immunofluorescence and flow cytometry analysis, and the cells were confirmed to be mycoplasma-free before use. Mouse neural stem cells (Merck, SCR031) were cultured under the same conditions as NPCs and were used as controls or for the fabrication of NPCbots only in the in vivo mouse treatment experiments.

### Fabrication and characterization of NPCbots

Microfluidic chips were fabricated using a replica moulding technique. Initially, master moulds were created on silicon wafers using photolithography techniques and wet etching, utilizing a negative photoresist (SU-8 3010, micro resist technology). Following this, polydimethylsiloxane (PDMS) slabs were prepared over the silicon master moulds through the replica moulding process. For this step, 10:1 PDMS:curing agent (The Dow Chemical Company) mixture was prepared and degassed in the desiccator for 30 min. Then, the mixture was poured over the silicon master moulds and cured in the oven at 60 °C for 45 min. After the curing process, PDMS slabs were precisely cut and punched to establish inlet and outlet holes utilizing a 1.5-mm biopsy puncher. Subsequently, these PDMS chips were irreversibly bonded to a PDMS-coated glass slide via oxygen plasma activation to complete the fabrication process.

During the microrobot fabrication phase, cells were isolated within the traps located in the trapping chamber of the LoC device. This isolation was achieved via flushing the growth medium solution including NPCs (at a concentration of 5 × 10^5^ cells ml^−1^) at a flow rate of 1 µl min^−1^ for 20 min, which was then followed by the flow of magnetoelectric nanoparticle solution in growth medium (33 mg ml^−1^) for in situ NPCbot incubation. After the 30-min incubation period, the outlet of the LoC device was reconfigured as an inlet for the reverse flow (growth medium at 15 µl min^−1^), facilitating the release of the incubated NPCbots from the traps, whereas the three inlets were utilized as outlets for collecting the NPCbots. This bidirectional design of the LoC device enhances the efficiency of the trapping and release mechanisms, thereby supporting the mass production and expedited in situ fabrication of biohybrid microrobots, that is, NPCbots. All the experimental procedures were meticulously documented using an Olympus IX73 microscope.

To assess the viability of the NPCbots, we used live/dead staining. An NPC cell stock solution, at a concentration of 1,000 cells μl^−1^, was incubated with CFO–BTO nanoparticles at varying concentrations (0, 500, 1,000, 1,500 and 2,000 ng μl^−1^) to obtain different concentrations of NPCbots. After 48 h, the viability was subsequently evaluated using the Live/Dead Cell Imaging Kit (488/570, Invitrogen). This kit utilizes a combination of Live Green and Dead Red components, which were mixed and incubated for 15 min at room temperature. Fluorescence images were captured using a confocal microscope (LSM 880, Carl Zeiss) and analysed using ImageJ (v. 2.3.0) software. This procedure facilitated the determination of an optimized CFO–BTO dosage, which was then used to ascertain the ideal incubation time needed to maintain the nanoparticles on the cell surface, thereby minimizing cellular uptake.

To further investigate the cellular uptake of CFO–BTO nanoparticles, we labelled them with Alexa Fluor 594. This labelling involved a two-step surface modification process. First, CFO–BTO nanoparticles were coated with APTES to introduce amino groups on their surface. This was performed by dispersing the nanoparticles in a 5% (v/v) APTES ethanol solution and allowing the reaction to proceed with continuous stirring at room temperature for 2 h. Post-APTES coating, the nanoparticles were centrifuged at 10,000*g* for 10 min, washed three times with ethanol and redispersed in PBS (pH 7.4). Fourier transform infrared spectroscopy was used for characterization. For the conjugation of Alexa Fluor 594, the APTES-coated nanoparticles were incubated with Alexa Fluor 594 NHS Ester (succinimidyl ester) at a concentration of 0.1 mg ml^−1^ in PBS. This step, conducted at room temperature for 1 h under gentle agitation, allowed the NHS ester to react with the amine groups to form stable amide bonds. The nanoparticles were subsequently centrifuged, washed three times with PBS to remove excess dye and resuspended in PBS. They were stored at 4 °C and protected from light until further use. To quantitatively assess the cellular uptake, dye-labelled CFO–BTO nanoparticles were incubated with cells for 0.25, 0.5, 1 and 2 h. Following incubation, cells were washed eight times with cold PBS and analysed using flow cytometry (LSRFortessa, BD Biosciences).

A Hitachi SU5000 field-emission SEM device was used to examine the morphology of the NPCbot. Additionally, the distribution of CFO–BTO on the NPCbot surface was analysed using the Zeiss LSM 880 with *z*-stack analysis.

### Stimulation of NPCbots by an AMF

For the assessment of the differentiation of NPCbots, the samples were subjected to an AMF stimulation protocol. AMFs were applied using a solenoid coil with an inside diameter of 5 cm in which the samples were placed. The solenoid was driven in a resonant tank configuration and generated a magnetic field with an amplitude of 20 mT and frequency of 1.18 kHz at its centre. Air cooling was used to stabilize the temperature of the solenoid. Each sample received AMF stimulation for 2 h, twice daily over a 2-day period. Following stimulation, the cells were fixed using 4% paraformaldehyde for 10 min at room temperature. Permeabilization was facilitated by treating with 0.5% Triton X-100 for 30 min at room temperature. To minimize non-specific binding, the samples were blocked with 2% bovine serum albumin for 30 min. For the detection of neuronal markers, the cells were incubated overnight at 4 °C with primary antibodies directed against βIII-tubulin (Sigma-Aldrich, T2200), MAP2 (Proteintech, 67015-1), α-tubulin (Merck, T6793) and GFAP (Agilent, Z0334). This was followed by incubation with secondary antibodies: FITC-conjugated goat anti-rabbit IgG (Sigma-Aldrich, AP132F) and TRITC-conjugated goat anti-mouse IgG (Sigma-Aldrich, AP503R), each for 1 h. After secondary antibody application, the samples underwent several washes and were subsequently stained with nuclear markers. Before NPC cell bot fabrication, immunofluorescence was utilized to verify the specificity of NPC cell markers, including Nestin (Sigma, ABD69) and SOX2 (Sigma, MAB4343), using the aforementioned secondary antibodies. For the inhibitor experiments, the culture medium was supplemented with lanthanum chloride at a concentration of 50 µM. It is important to note that the inhibitor was introduced to the culture medium before the commencement of the stimulation experiments. For the investigation of intracellular Ca^2+^ influx, NPC spheroids or NPCbots were subjected to the described stimulation for durations of 2 h or 4 h. Subsequently, these cells were stained with the Fluo-4 AM probe (Abcam, ab241082) for 20 min. The stained samples were visualized using a Zeiss LSM 880 Airyscan confocal laser scanning microscope. We used the Cellpose algorithm for cellular segmentation, followed by quantitative analysis using ImageJ software^46^. To ascertain the differentiation percentage of NPCbots, samples were subjected to identical stimulation protocols for 2 h, administered twice daily across either a 2-day or a 7-day period. In particular, the NPC stimulation reflects AMF induction under NPC expansion conditions, without applying any standard differentiation protocol. Post-stimulation, the cells were stained using Alexa Fluor 488 βIII-tubulin antibody (Biolegend, catalogue number 801203) or Alexa Fluor 488 GFAP antibody (BD Biosciences, catalogue number 561449). The stained samples were subsequently analysed via flow cytometry using an LSRFortessa instrument (BD Biosciences). For the electrophysiological measurements, samples were subjected to identical stimulation protocols for 2 h, administered twice daily across either a 7-day or a 14-day period. Post-stimulation, voltage recordings were conducted following established protocols, as previously described^47^. These measurements were performed using conventional patch-clamp techniques in a whole-cell configuration, utilizing an Axopatch 200 amplifier. Data acquisition was facilitated through a Digidata 1322A interface (Molecular Devices), with the amplifier interfaced to a computer running Clampex (v. 10.2) software.

### Long-term biocompatibility under AMF stimulation

To evaluate the long-term cytotoxicity of the optimized dose of CFO–BTO (1 ng per cell), the NPCbots were subjected to a series of assessments over time points of 0.5, 1, 2, 7 and 14 days. These microrobots underwent identical stimulation protocols for 2 h, administered twice daily. NPCbots were seeded at a density of 1 × 10^4^ NPCbots per well in a 12-well chamber slide (ibidi, catalogue number 81201). For cell viability assessment, the 3-(4,5-dimethylthiazol-2-yl)-2,5-diphenyltetrazolium bromide assay was conducted using a concentration of 3 mg ml^−1^. Absorbance was measured at a wavelength of 570 nm with a reference wavelength of 630 nm using the Varioskan Flash multimode microreader. The mean absorbance value was calculated based on measurements obtained from triplicate wells.

### Western blot analysis

Protein extraction from cells, following exposure to an a.c. magnetic field, was conducted using Thermo Scientific RIPA Lysis and Extraction Buffer (catalogue number 89900), which was supplemented with PMSF Protease Inhibitor (catalogue number 36978) to ensure comprehensive protein recovery and inhibition of protease activity. Protein concentrations were determined using the Thermo Scientific Pierce BCA Protein Assay Kit (catalogue number 23227). Proteins were subsequently resolved by electrophoresis on sodium dodecyl sulfate–polyacrylamide gel electrophoresis gels, prepared using Tris-Glycine-SDS Buffer (Sigma, T7777) at the appropriate working concentration. Post-electrophoresis, proteins were transferred onto polyvinylidene fluoride membranes using Thermo Scientific Pierce 10X Western Blot Transfer Buffer, Methanol-free (catalogue number 35040). To prevent non-specific binding, membranes were blocked with Thermo Scientific Pierce Clear Milk Blocking Buffer (10X), diluted to a 1× concentration (catalogue number 37587) and incubated for 1 h at room temperature. Membranes were then incubated overnight at 4 °C with primary antibodies: Anti-beta III-Tubulin (Abcam, ab18207), Anti-alpha Tubulin (Abcam, ab52866), Anti-GFAP (Abcam, ab68428) and Anti-MAP2 (Abcam, ab32454), all diluted following the manufacturer’s recommendations. After primary antibody incubation, membranes were washed and incubated with HRP-conjugated Affinipure Goat Anti-Rabbit IgG(H + L) (Proteintech, SA00001-2) for 1 h at room temperature. The detection of protein bands was achieved using Clarity Western ECL Substrate (Biorad, 200 ml number 1705060) and visualized on an Amersham Imager 600 system, which was equipped with ImageQuant TL (v. 8.1) software for digital imaging and quantitative analysis.

### Zebrafish care

Zebrafish (*Danio rerio*) were maintained under a standard 14-h light/10-h dark cycle at a water temperature of 28 °C (ref. ^48^). The zebrafish lines used in this study included wild-type WIK, Tg(kdrl:mCherry)^49^ and Tg(kdrl:mCherry);Tg(gfap:GFP)^50^. Embryos were raised in E3 medium (5 mM of NaCl, 0.17 mM of KCl, 0.33 mM of CaCl_2_ and 0.33 mM of MgSO_4_) supplemented with either 0.01% methylene blue to inhibit fungal growth and/or 0.2 mM of 1-phenyl-2-thiourea (Sigma-Aldrich) to prevent pigment development.

### Manipulation of NPCbots via magnetic actuation

A magnetic manipulation apparatus, facilitating five-degrees-of-freedom wireless micromanipulation, was utilized for evaluating NPCbot swimming capabilities (MFG-100, MagnebotiX) and used to control the NPCbot’s trajectory. These assessments were conducted in a PBS solution using a rotating magnetic field to propel the NPCbots. Movements were documented using an Olympus IX81 fluorescence microscope.

For in vivo manipulation within zebrafish larvae, NPCbots were prelabelled using live dye (L3224, LIVE/DEAD Viability/Cytotoxicity Kit, Thermo Fisher Scientific) as per the manufacturer’s instructions. The NPCbots were then introduced into the duct of Cuvier in zebrafish embryos (2 dpf, kdrl:mCherry line) embedded in low-melting-point agarose, using a FemtoJet injection system (Eppendorf). Each injection delivered a volume of 2–3 nl of NPCbots (200–300 NPCbots). Consistency in the amount of injected NPCbots was ensured by using the same injection capillary for all larvae, with the process being monitored under a Leica M205 FCA stereomicroscope. Post-injection, the zebrafish embryos were carefully extracted from the original embedding medium and re-embedded in fresh low-melting agarose.

Magnetic control during in vivo experiments was achieved by applying a rotating magnetic field supplemented by a compensatory magnetic field gradient to counteract blood flow. The in vivo movements of NPCbots were recorded using a Leica M205 FCA stereomicroscope. Positional data were abstracted and analysed using Imaris software (v. 9.9, Bitplane), facilitating the calculation of swimming speeds, temporal data and trajectory plotting of the NPCbots.

### Evaluation of NPCbots in vivo therapeutic performance on SCI in a zebrafish larvae model

Following the assessment of NPCbot’s mobility, its therapeutic efficacy was evaluated using a zebrafish SCI model. The NPCbots were injected at the site of injury and subjected to an AMF. To establish the SCI model in zebrafish larvae (wild-type WIK line), we anaesthetized the larvae (2 dpf) in E3 medium supplemented with 0.02% Tricaine (ethyl 3-aminobenzoate methanesulfonate, MS-222). The anaesthetized larvae were then transferred to a surgery plate. Excess E3 medium was carefully removed using an aspiration pipette. Positioned laterally on the plate, the larvae were approached dorsally with a microscalpel (30 G, three 1/2-inch hypodermic needles, BD Biosciences, catalogue number 305106) modified by removing the barrel flange and attaching a cell scraper handle^44^. The incision was made across the dorsal trunk from the notochord to the dorsal edge, with the needle’s bevel oriented sideways. Lesions were standardized to approximately two myotomes in diameter (around 250 μm) and ensured that the notochord remained intact. These lesions were carried out under an optical microscope to confirm the complete excision of spinal cord tissue at the lesion site, maintaining consistency in the lesion location across all specimens. After lesioning, the larvae were gently transferred into a recovery dish using a pipette filled with E3 medium and subsequently placed in an incubator maintained at 28 °C for recovery. The consistent incision procedure was used for the 2-dpf zebrafish larvae of the kdrl:mCherry line, facilitating the live-imaging process. The NPCbots were injected into the injury site, using 200–300 ng of CFO–BTO and 200–300 NPCs as a control, followed by the application of an AMF. Magnetic treatments were administered over a 2-h period, with additional treatments provided on 3 and 4 dpf. Post-injection, the larvae were incubated at 34 °C.

The therapeutic efficacy of NPCbots at the molecular level was examined through whole-mount embryo immunohistochemistry. Larvae were fixed in 4% formaldehyde in PBS, and subsequently washed in PBS with 0.5% Tween and 0.5% Triton X-100 (PBST). To bleach pigments in whole-mount larvae, we rehydrated the larvae in a stepwise manner with 75%, 50% and 25% methanol in PBST for 5 min each. We rinsed twice with PBST for 5 min each. A 5 ml of bleaching solution was prepared using 4,425 µl of ddH2O, 125 µl of 20× saline-sodium citrate buffer, 250 µl of formamide and 200 µl of 30%–35% peroxide. The embryos were incubated in this solution for about 1.5 h under direct light or in a light box covered with aluminium foil, checking regularly. After bleaching, we rinsed twice with PBST for 5 min each. Proteinase K treatment was conducted at a concentration of 10 µg ml^−1^ in PBST for 30 min at 37 °C, followed by a series of PBST washes and a post-fixation step in formaldehyde for 30 min at room temperature. Embryos were then treated with cold acetone at –20 °C for 20 min, washed in PBST and incubated in freshly prepared blocking solution (5% normal goat serum, 1% bovine serum albumin and 1% dimethyl sulfoxide in PBST) at room temperature for 2 h. Primary antibodies specific for zebrafish GFAP (Agilent, catalogue number Z0334) and α-tubulin (Sigma-Aldrich, catalogue number T6793) were diluted 1:300 in the blocking solution and the embryos incubated overnight at 4 °C. Following this, embryos were washed in PBST and incubated overnight at 4 °C in the dark with secondary antibodies, Alexa Fluor 488 anti-rabbit (Thermo Fisher Scientific, catalogue number A32731) and Alexa Fluor 555 anti-mouse (Thermo Fisher Scientific, catalogue number A11008), both at a dilution of 1:300. After final washes in PBST, the embryos were embedded in 1% low-melting-point agarose, positioned in confocal culture dishes (Thermo Fisher Scientific) and imaged using a Zeiss LSM 880 confocal laser scanning microscope. Image processing was performed using ImageJ (v. 2.3.0) software.

To investigate whether NPCbots contribute to natural neural regeneration, live-imaging techniques were used. Specifically, the GFAP-transgenic zebrafish line expressing GFP was utilized. Following SCI induction at 2 dpf, NPCbots (200–300 bots per specimen) were administered, with native NPCs serving as a control. Subsequent treatment involved the application of a uniform a.c. magnetic field for 2 h per session on 2, 3 and 4 dpf. Post-treatment, the zebrafish larvae were incubated at 34 °C. Imaging was performed using the LSM 880 confocal microscope, and the colocalization analysis was conducted using ImageJ software.

To further investigate the behavioural responses of zebrafish during our therapeutic intervention, we conducted assessments on six distinct groups that received treatment. The behavioural monitoring was executed using a Zebrabox (ViewPoint Life Sciences). Each zebrafish larva was individually placed in a compartment within a 96-well plate, specifically designed to provide 16 separate Zebrabox environments. Observations were made over a 15-min interval during which the larvae were subjected to alternating light and dark phases, each lasting 5 min. The movement of each larva was quantitatively recorded every second. All behavioural assessments were consistently performed within the same time period, specifically between 2 p.m. and 6 p.m., at 1 and 3 dpi. The video footage captured during these sessions was subsequently analysed with EthoVision XT11 (v. 11.5) software (Noldus Information Technology), which facilitated the generation of heat maps that depicted the positional distribution of the zebrafish over time.

### SCIs in mice and NPCbot transplantation

In this study, 8-week-old male C57BL/6 mice, weighing 18–22 g, were utilized to establish a model of SCI. Each group was assigned ten mice, with five undergoing motor function evaluation and the other five undergoing histological and physiological assessments. Initially, anaesthesia was induced using 5% isoflurane delivered in oxygen at a flow rate of 2 l min^−1^. Maintenance of anaesthesia was achieved with 1% isoflurane in oxygen at a reduced flow of 1 l min^−1^. Before surgery, the fur on the mouse’s back was shaved to expose the operative site, which was subsequently sterilized using povidone–iodine. A vertical midline incision was made at the T10 vertebral level with a surgical scalpel, and the skin was retracted. Blunt dissection was used to separate the subcutaneous tissues and fascia, revealing the spinous processes. Subsequently, the paraspinal muscles were dissected to expose the laminae, which were then bilaterally opened to access the spinal cord. A complete removal of 2-mm segments of the spinal cord was performed. The surgical site was closed in layers—muscles, subcutaneous tissue, fascia and skin. After a 7-day recovery period, a solution containing 5 × 10^5^ NPCbots suspended in 5 μl of fibrin gel was prepared. This gel was composed of 25 mg ml^−1^ of fibrinogen (MB5809, Dalian Meilun Biotech) and 25 U ml^−1^ thrombin (MB1368, Dalian Meilun Biotech), and was immediately transplanted into the lesion epicentre using a Neuros Syringe (Hamilton). For control experiments, 0.5 mg of CFO–BTO or 5 × 10^5^ NPCs were used and also injected at the injury site with the fibrin gel. In the experimental group receiving magnetic guidance, cylindrical neodymium–iron–boron magnets were positioned at the injury site to facilitate the orientation of NPCbots for 30 min. This was followed by the application of an AMF for 30 min daily, from day 7 to day 21 post-surgery. For postoperative management, cefuroxime was administered for 7 days, and manual bladder expression was performed twice daily to assist in recovery.

### Behavioural studies in mice model of SCI

Motor function in mice with SCIs was evaluated using BMS. Assessments were conducted by independent observers who were blinded to the experimental conditions on 7, 21 and 34 dpi. Each mouse was observed for a period of 8 min and scored independently by the observers. On day 34, locomotor activity and gait patterns were further analysed using a walking track assay. Mice traversed a custom-built narrow Plexiglass trough (5 cm wide and 35 cm long), during which their movements were recorded on video. Before the assay, the forelimbs and hindlimbs were marked with non-toxic paint (blue and red, respectively), leaving identifiable paw prints on a white sheet that lined the trough. These prints were subsequently scanned and digitized images were analysed using ImageJ software. Measurements were taken from at least five footprints per side, collected over three separate trials per animal, and average values were computed. Additionally, on day 34, a behaviour observation box (30 cm × 20 cm × 20 cm) was used to assess motility and area preference. Overhead videos were recorded for 8 min and analysed using EthoVision XT11 software (Noldus Information Technology). This analysis generated trajectory and heat maps, illustrating the spatial distribution and movement patterns of the mice over time. Functional recovery on day 34 was also evaluated using the toe spread test. During this test, mice were gently suspended by the tail, and the extent of hindlimb toe spreading was observed as the animals attempted to escape. Toe spread was scored on a five-tier scale: 0 indicated no visible toe spread, 1 represented minimal spreading with one toe extended, 2 was assigned when two toes were extended, 3 indicated all toes were separated but less than normal and 4 corresponded to a normal toe spread.

### Immunohistochemistry

On day 35, tissues were embedded in paraffin and sectioned to a thickness of 7 µm. These sections were then deparaffinized in xylene and rehydrated through a series of graded alcohol baths. Antigen retrieval was performed using sodium citrate buffer. For immunolabeling, the tissue sections were incubated with primary antibodies against βIII-tubulin (Abcam, ab78078) and GFAP (Abcam, ab7260) overnight at 4 °C. This was followed by incubation with the secondary antibodies, Goat Anti-Mouse IgG H&L conjugated with Alexa Fluor 647 (Abcam, ab150115) and Goat Anti-Rabbit IgG H&L conjugated with Cy3 preadsorbed (Abcam, ab6939), for 1 h at 37 °C. Nuclei were stained using DAPI. The stained samples were subsequently examined under a confocal microscope (Leica), and the colocalization analysis was conducted using ImageJ software.

### MEP analysis

To evaluate neuronal connectivity across the lesion site within the SCI model, MEPs were recorded on 35 dpi. The mice underwent anaesthesia with 1.25% of 2,2,2-tribromoethanol administered at a dosage of 0.2 ml per 100 g body weight. Subsequently, a craniotomy was conducted to expose the primary motor cortex (M1) area. Utilizing a stereotaxic device, a stimulation electrode was precisely positioned within the sensorimotor cortex at a depth of 800–900 μm from the cerebral surface to specifically target corticospinal neurons. For the measurement of MEPs, a recording electrode was affixed to the gastrocnemius muscle, and a ground electrode was secured to the tail. Stimulation was delivered as a single square-wave pulse with an intensity of 0.5 mA and duration of 0.5 ms, followed by a 2-ms delay, at a frequency of 1 Hz. The MEP peak amplitude was quantified as the voltage difference between the prestimulation baseline and the maximum of the initial response wave. All signals were amplified and captured using a digital oscilloscope (Anhui Yaokun Biotechnology).

### Safety assessment

To evaluate the systemic safety, we assessed plasma cytokine levels using a Luminex-based assay. Peripheral blood was collected on day 35, followed by centrifugation at 500*g* for 10 min. The resultant supernatants were transferred to fresh tubes and stored at –80 °C until further analysis. The samples were then diluted according to the specifications of the Luminex assay buffer as per the manufacturer’s instructions. Heat maps of the cytokine levels were created using Heml software (v. 1.0.3.7). Body weight was monitored at 7-day intervals. On day 35, additional blood samples were obtained for the analysis of hepatic and renal functions. The major organs, including the heart, liver, spleen, lungs and kidneys, were harvested and subsequently fixed in formalin for histological examination with haematoxylin and eosin staining (Servicebio).

### Statistics and reproducibility

No statistical methods were used to predetermine the sample sizes, but our sample sizes are similar to those reported in previous publications. Data distribution was assumed to be normal but this was not formally tested. All animals or data points were included in the final analysis. For all animal studies, subjects were randomly assigned to experimental groups. BMS scores were evaluated by independent observers blinded to the treatment conditions. Although blinding was not feasible during the execution of in vitro studies due to procedural requirements, data analysis remained unbiased. All quantitative assessments were performed blinded to group allocation, with samples only being re-identified for final visualization and statistical testing. All results were reproduced at least three times; all data were indicated as mean ± s.d. or mean ± s.e.m. All statistical analyses were conducted using the Prism software package (PRISM 9.5.0; GraphPad software, 2022). *P* < 0.05 indicated statistical significance.

### Reporting summary

Further information on research design is available in the Nature Portfolio Reporting Summary linked to this article.

## Online content

Any methods, additional references, Nature Portfolio reporting summaries, source data, extended data, supplementary information, acknowledgements, peer review information; details of author contributions and competing interests; and statements of data and code availability are available at https://doi.org/10.1038/s41563-026-02625-3.

## Supplementary information

Supplementary Information Supplementary Notes 1 and 2, Figs. 1–18, captions to Supplementary Videos 1–5 and refs. 1–7.

Reporting Summary

Supplementary Video 1 Representative walking gait recordings of mice at 7, 21 and 34 days after various treatments. Scale bar, 3 cm.

Supplementary Video 2 Representative optical microscopy video showing magnetic manipulation of an NPCbot in a PBS solution. Scale bar, 10 μm.

Supplementary Video 3 Representative fluorescence microscopy video showing magnetic-field-driven manipulation of multiple NPCbots in a PBS solution. The NPCbots were labelled with live dye in green. Scale bar, 20 μm.

Supplementary Video 4 Representative stereomicroscopy video showing the magnetic manipulation of NPCbots in the DA of zebrafish larvae. The NPCbots, labelled with live dye in green, are shown navigating both with and against the blood flow. This was achieved using a 50-mT rotating magnetic field and a 700-mT m^−1^ compensatory magnetic field gradient to counteract hemodynamic forces. Scale bar, 10 μm. The video is played at 4× speed.

Supplementary Video 5 Representative stereomicroscopy video showing the magnetic manipulation of NPCbots in the pericardial cavity of zebrafish larvae. The manipulation was achieved using a 50-mT magnetic field and a 700-mT m^−1^ magnetic field gradient. The video is played at 2× speed.

Supplementary Data 1 Statistical source data for the Supplementary Figures.

## Source data

Source Data Fig. 2 Statistical source data.

Source Data Fig. 3 Statistical source data.

Source Data Fig. 4 Statistical source data.

Source Data Fig. 5 Statistical source data.

Source Data Fig. 6 Statistical source data.

Source Data Extended Data Fig. 1 Statistical source data.

## Data Availability

All relevant data supporting the findings of this study are available within the article and its Supplementary Information. Source data are provided with this paper.
