# Supplementary material for: Magnetoelectric microrobots for spinal cord injury regeneration
Source: Nat Mater. 2026 Jun 2;25(8):1458–69. doi: 10.1038/s41563-026-02625-3 (PMC13658017; doi:10.1038/s41563-026-02625-3)
Supplement: Supplementary file 1 [file 41563_2026_2625_MOESM1_ESM.pdf]

nature materials

Supplementary information

https://doi.org/10.1038/s41563-026-02625-3

Magnetoelectric microrobots for spinal cord injury regeneration

In the format provided by the authors and unedited

Table of Contents

Supplementary Videos Legends 2
- Supplementary Video 1. 2
- Supplementary Video 2. 2
- Supplementary Video 3. 2
- Supplementary Video 4 2
- Supplementary Video 5 2

Supplementary Notes 3
- Supplementary Note 1 3
- Supplementary Note 2 4

Supplementary Figures 6
- Supplementary Figure 1 6
- Supplementary Figure 2 7
- Supplementary Figure 3 8
- Supplementary Figure 4 9
- Supplementary Figure 5 10
- Supplementary Figure 6 11
- Supplementary Figure 7 12
- Supplementary Figure 8 13
- Supplementary Figure 9 14
- Supplementary Figure 10 15
- Supplementary Figure 11 16
- Supplementary Figure 12 17
- Supplementary Figure 13 18
- Supplementary Figure 14 19
- Supplementary Figure 15 20
- Supplementary Figure 16 21
- Supplementary Figure 17 22
- Supplementary Figure 18 23

Supplementary References 24

Supplementary Videos Legends

Supplementary Video 1. Representative walking gait recordings of mice at 7, 21 and 34 days after various treatments. Scale bar, 3 cm.

Supplementary Video 2. Representative optical microscopy video showing magnetic manipulation of an NPCbot in PBS solution. Scale bar, 10 μm.

Supplementary Video 3. Representative fluorescence microscopy video showing magnetic-field-driven manipulation of multiple NPCbots in PBS solution. The NPCbots were labeled with live dye in green. Scale bar, 20 μm.

Supplementary Video 4. Representative stereomicroscopy video showing magnetic manipulation of NPCbots in the dorsal aorta of zebrafish larvae. The NPCbots, labelled with live dye in green, are shown navigating both with and against the blood flow. This was achieved using a 50 mT rotating magnetic field and a 700 mT/m compensatory magnetic field gradient to counteract hemodynamic forces. Scale bar, 10 μm. The movie is played at 4× speed.

Supplementary Video 5. Representative stereomicroscopy video showing magnetic manipulation of NPCbots in the pericardial cavity of zebrafish larvae. The manipulation was achieved using a 50 mT magnetic field and a 700 mT/m magnetic field gradient. The movie is played at 2× speed.

Supplementary Notes

Supplementary Note 1

Mechanistic Insights into AMF-Induced NPCbot differentiation

To elucidate the molecular mechanisms underlying NPCbot-induced neural repair, we employed Western blotting to quantitatively assess protein expression levels of key neuronal and astrocytic markers at the cellular level. βIII-Tubulin and MAP2, pivotal for the structural integrity and connectivity of neurons, were examined. βIII-Tubulin serves as an indicator of neuronal formation and growth, while MAP2 enhances the stability and linkage among neuronal branches. Our results demonstrated significant increases in these proteins of NPCbots group under an AMF, consistent with enhanced neuronal differentiation (**Supplementary Figure 7a-f**). These molecular changes are indicative of the potential for improved motor functions once translated to an *in vivo* model. Further, we analyzed the expression of GFAP to understand the response of astrocytes – supportive glial cells essential for neural repair. The expression levels of GFAP indicated that the NPCbot group optimally activates astrocytes to aid in neural repair. Additionally, α-Tubulin, known for its role in maintaining neuronal shape and intracellular transport, showed increased abundance in the NPCbot group under AMF (**Supplementary Figure 7g-l and Supplementary Figure 18**). This suggests that our treatment supports the structural rebuilding and functional improvement of neurons, potentially contributing to enhanced locomotive abilities once applied *in vivo*.

Under AMF, the magnetostrictive CFO core within the CFO-BTO nanoparticles undergoes mechanical strain. This strain is transferred to the BTO shell, inducing a transient alteration in the surface charges of the nanoparticles. During this process, generation of heat and mechanical force may occur[32, 46]. Theoretically, pure CFO is expected to produce more heat and mechanical force compared to CFO-BTO[33, 47]. However, immunofluorescence and quantitative analyses reveal that CFO alone does not significantly promote differentiation under AMF (**Supplementary Figure 8**), further substantiating that the primary mechanism by which CFO-BTO influences NPC differentiation is through charge-mediated stimulation.

To further elucidate the differentiation mechanisms of NPCs under NPCbot treatment, our research focused on the cAMP-dependent pathway, also known as the adenylyl cyclase (AC) pathway, which can be activated by various extracellular stimuli[48]. Elevated intracellular Ca^{2+} concentrations, whether directly or indirectly, form a complex with calmodulin (CaM), leading

to the activation of AC[49]. AC is a pivotal regulatory enzyme that catalyzes the conversion of ATP (Adenosine triphosphate) to cyclic AMP (cAMP). The second messenger cAMP activates the protein kinase A (PKA)-dependent pathway for neural progenitor cell differentiation[50]. Prior research indicates that electrical stimulation can activate intracellular Ca^{2+} channels, resulting in an increased influx of intracellular Ca^{2+}[43, 51]. As shown in **Supplementary Figure 9**, the magnetoelectric stimulations, as delivered by the AMF in the NPCbot, enhance calcium influx through these channels. Additionally, we introduced $LaCl_3$, a calcium channel blocker[52], into our experimental set-up. Utilizing confocal microscopy for in-depth analysis, we observed a marked decrease in the expression of both neuronal and astrocytic markers upon the addition of $LaCl_3$ (**Supplementary Figure 8c-h**). This finding underscores the crucial role of calcium channel activation in the AMF-dependent differentiation process facilitated by NPCbots. These insights not only provide a molecular basis for the observed enhancements in structural and functional cellular attributes but also substantiate the mechanistic efficacy of our NPCbot treatment.

Supplementary Note 2

Long-term Stimulation supports sustained viability and neuronal maturation of NPCbots

To investigate the effects of prolonged AMF stimulation on NPCbots, we employed the MTT assay to assess long-term biocompatibility. Results indicated that the NPCbots maintained over 80% viability throughout a 14 d stimulation period, with no obvious decrease in cell viability, underscoring their potential for sustained therapeutic use (**Supplementary Figure 10**). Further examination of our stimulation strategy was aimed at determining the extent of NPCbot differentiation into neurons and astrocytes. For this purpose, NPCbots were subjected to AMF stimulation for a duration of 7 d. Flow cytometry revealed a predominant differentiation of NPCbots into neurons, with approximately 80% of the NPCbots differentiating into neurons and 5% into astrocytes after 7 d (**Supplementary Figure 11 and Supplementary Figure 17**). This finding highlights the potential of NPCbots to enhance neuronal connections in vivo. Additionally, a 14 d stimulation regimen was applied to evaluate functional maturation of the derived neurons. Patch clamp analyses conducted post-stimulation revealed that approximately 29% of the NPCbots began to exhibit spontaneous action potentials, indicating neuronal activation (**Supplementary Figure 12**). Moreover, 46% of the NPCbots displayed oscillatory behaviors in their membrane potential, which, although not strong enough to qualify as full

action potentials, demonstrated greater activity compared to the baseline where 98% of the NPCbots showed no activity. These observations suggest successful neuronal differentiation and maturation in NPCbots-derived neurons under extended AMF stimulation.

Supplementary Figures

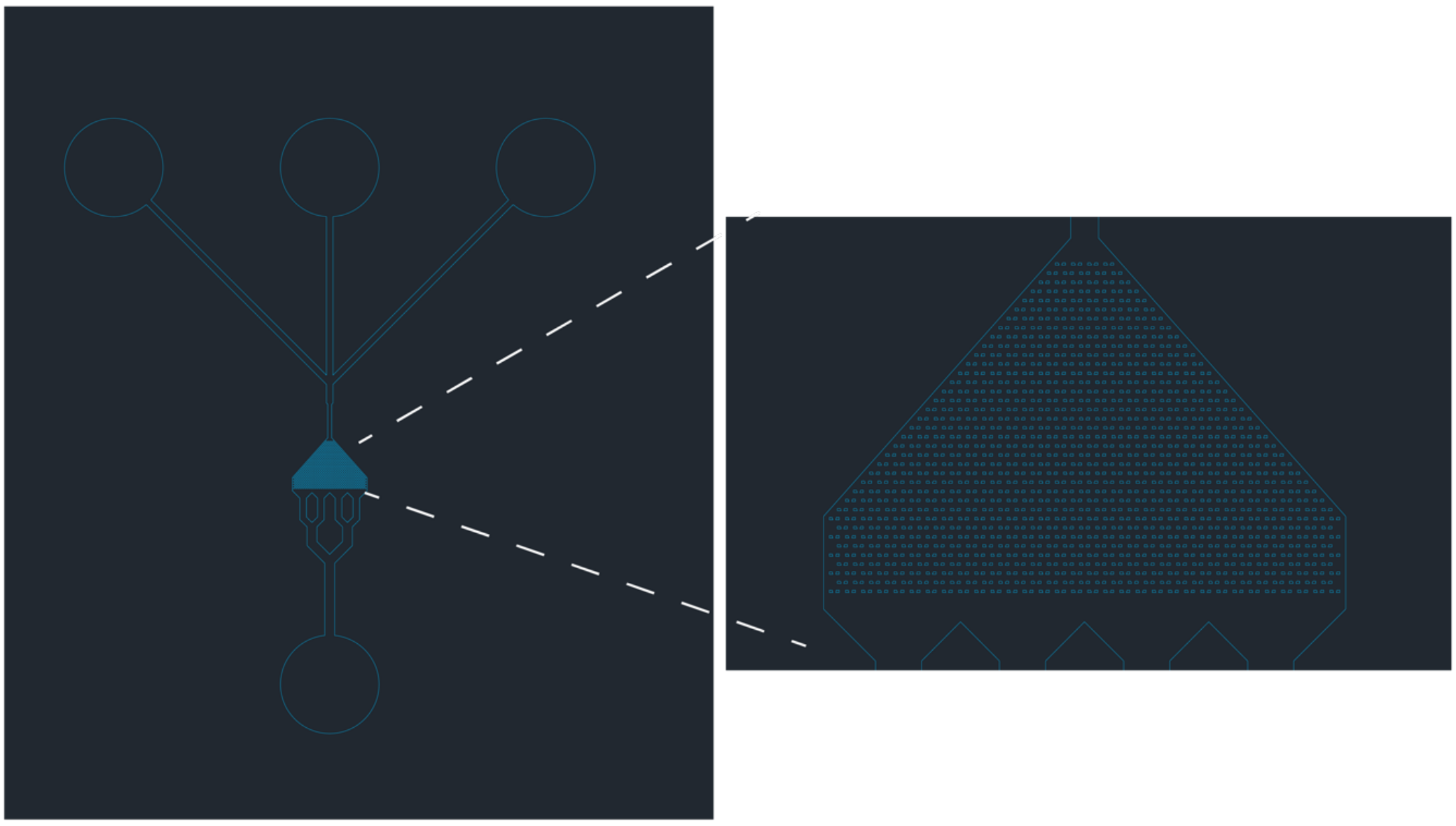

Supplementary Figure 1. Microfluidic device for NPCbots fabrication. This figure presents the schematic layout of a lab-on-a-chip system designed for precise cell manipulation and analysis. The inset provides a magnified view of the individual cell traps.

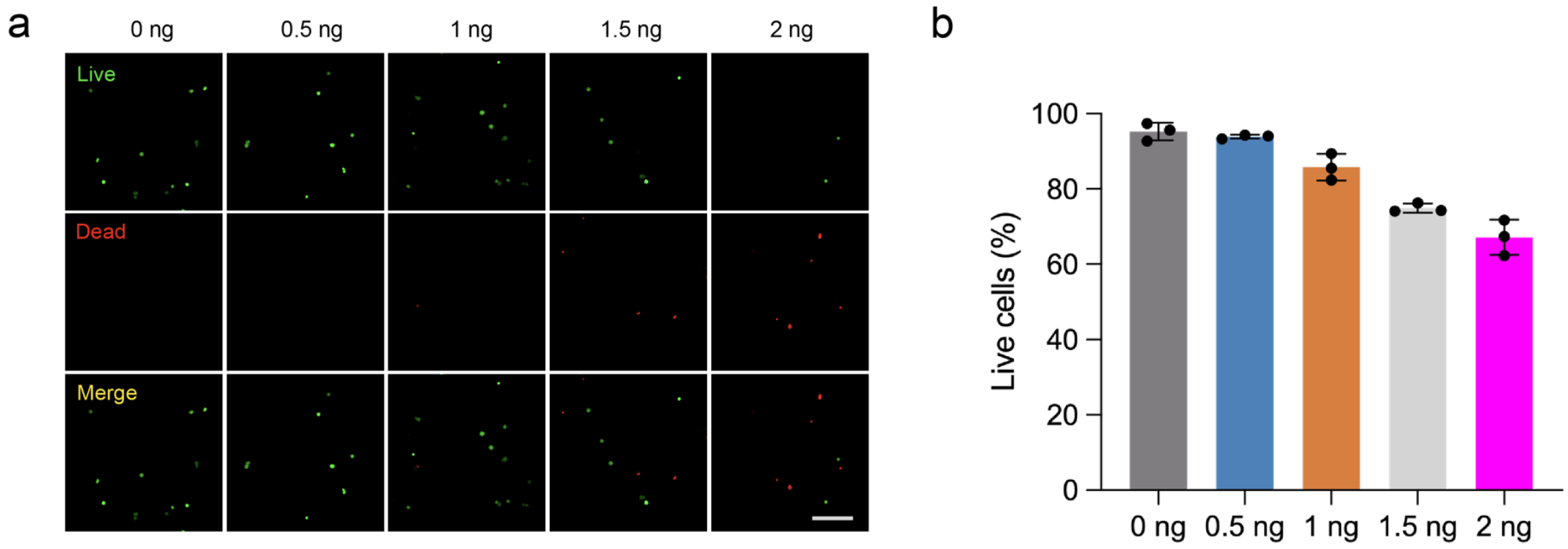

Supplementary Figure 2. Cell viability assessment of the fabricated NPCbots across varying doses of CFO-BTO at 48 h. a, Live/dead assay and (**b**) MTT assay to optimize the CFO-BTO nanoparticle dosage required for NPCbot fabrication, indicating cell viability (green: live; red: dead; scale bar = 50 μm). As shown in the figure, CFO-BTO doses up to 1 ng per cell showed good biocompatibility (n = 3 experiments, data are presented as mean ± s.d.).

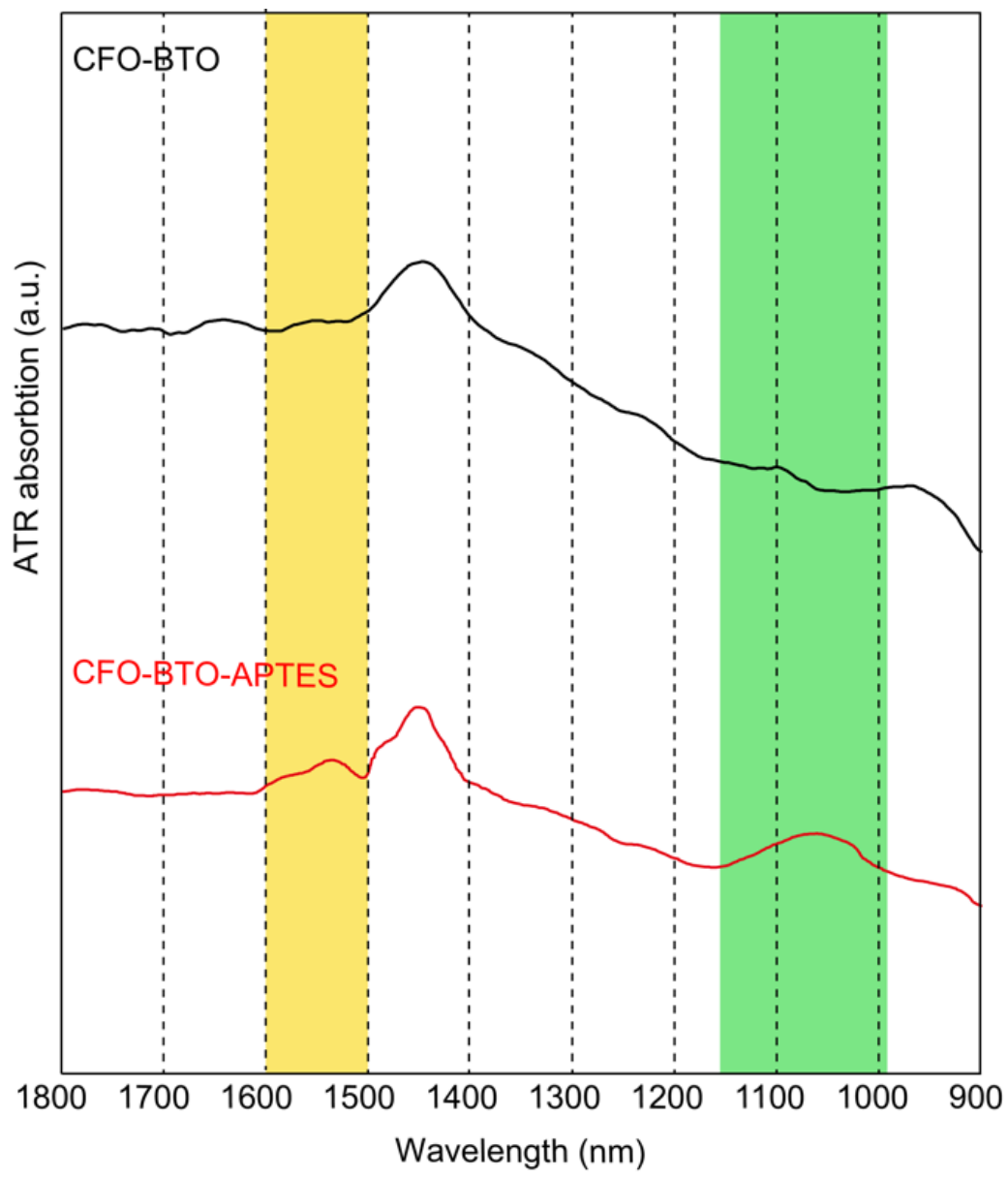

Supplementary Figure 3. FT-IR Spectra Analysis of Surface-Modified CFO-BTO. This figure presents the FT-IR spectra for both uncoated and (3-aminopropyl)triethoxysilane (APTES)-coated CFO-BTO. The yellow highlighted region indicates the presence of amine groups, suggesting successful surface modification of CFO-BTO nanoparticles through APTES conjugation. Additionally, the green highlighted region shows the Si-O-Si signal, characteristic of APTES, confirming its conjugation to the CFO-BTO nanoparticles.

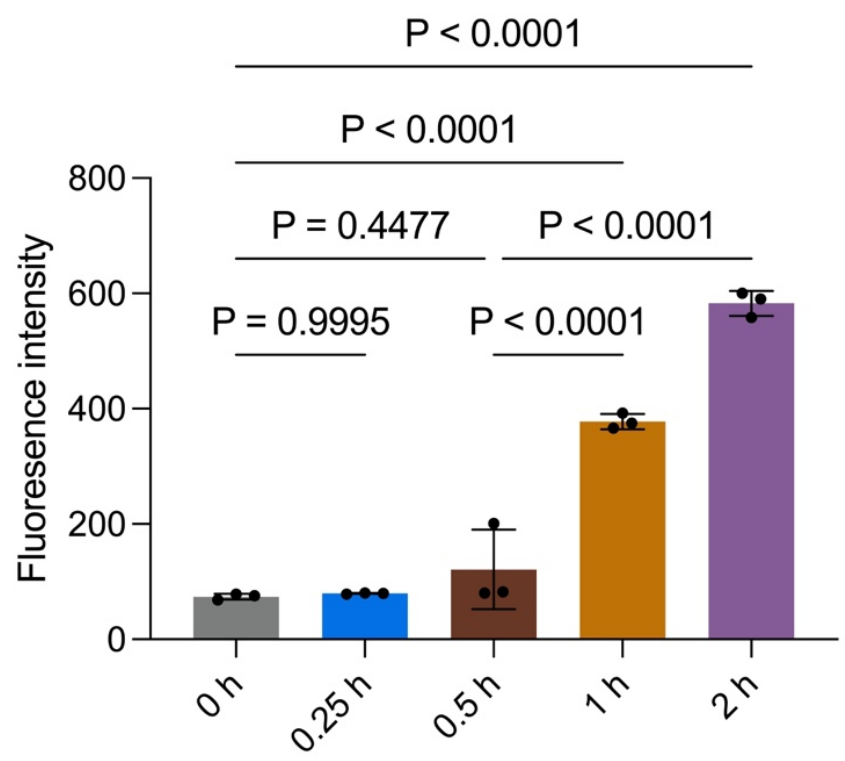

Supplementary Figure 4. Flow cytometry assays measuring the uptake of Alexa Fluor 594-conjugated CFO-BTO by NPCs following incubation periods of varying lengths. n = 3 experiments, data are presented as mean± s.d. Statistical significance was calculated via one-way ANOVA with a Tukey post hoc test.

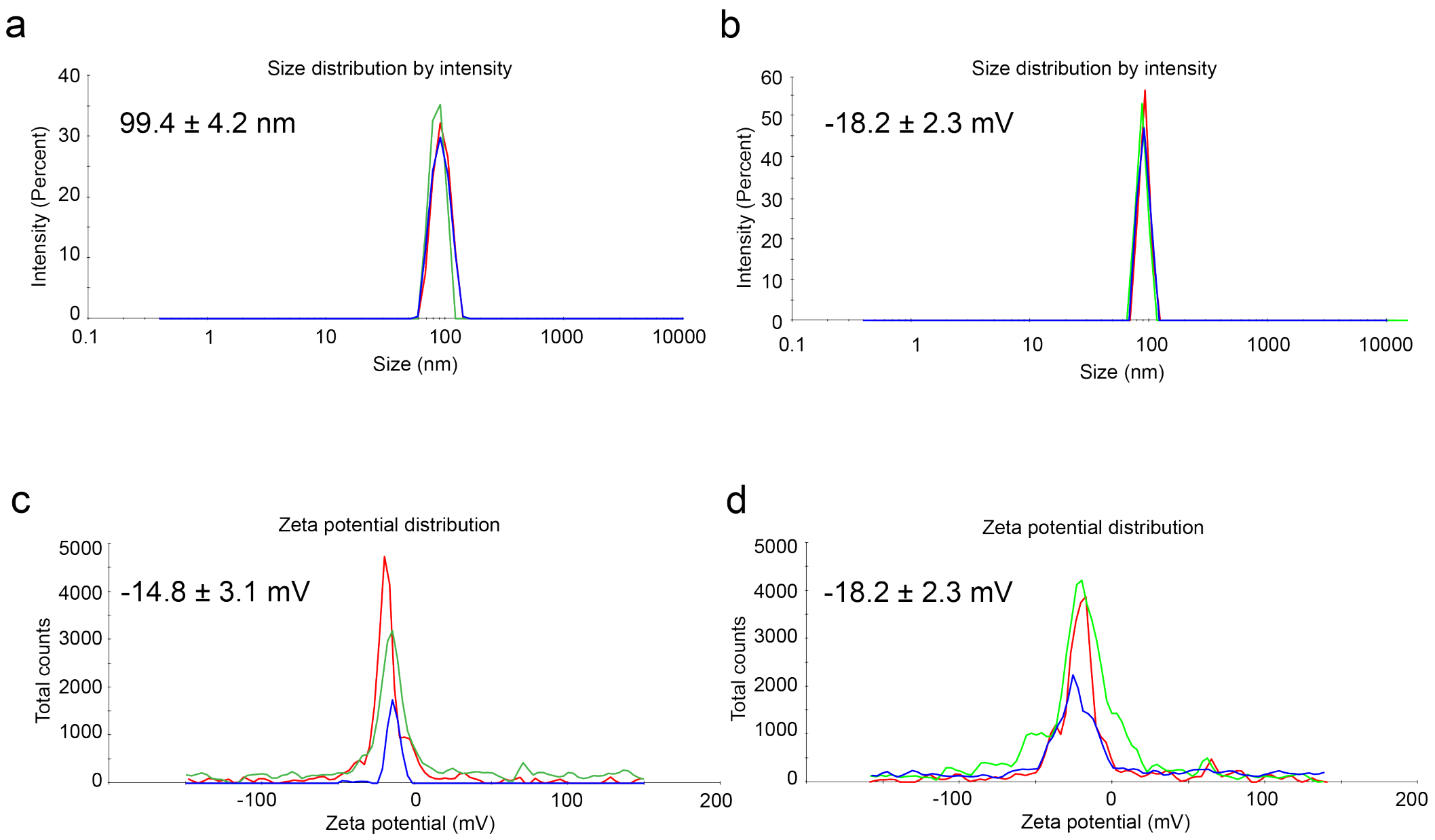

Supplementary Figure 5. Size and zeta potential distribution of CFO-BTO and Alexa Fluor 594-labeled CFO-BTO in PBS. a, Particle size distribution and (**c**) zeta potential measurements of CFO-BTO in PBS (n = 3). **b,** Particle size distribution and (**d**) zeta potential measurements of Alexa Fluor 594-labeled CFO-BTO in PBS (n = 3).

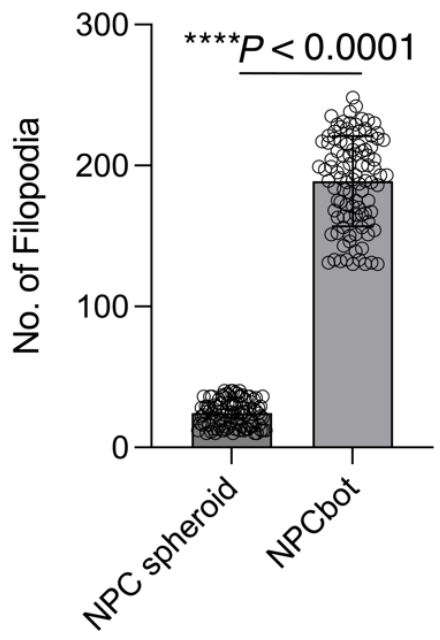

Supplementary Figure 6. Quantification of filopodia in NPC spheroids and NPCbots, assessed using scanning electron microscopy (SEM). n = 100 per group, data are presented as mean ± s.d. Statistical significance was calculated via Student's t-tests.

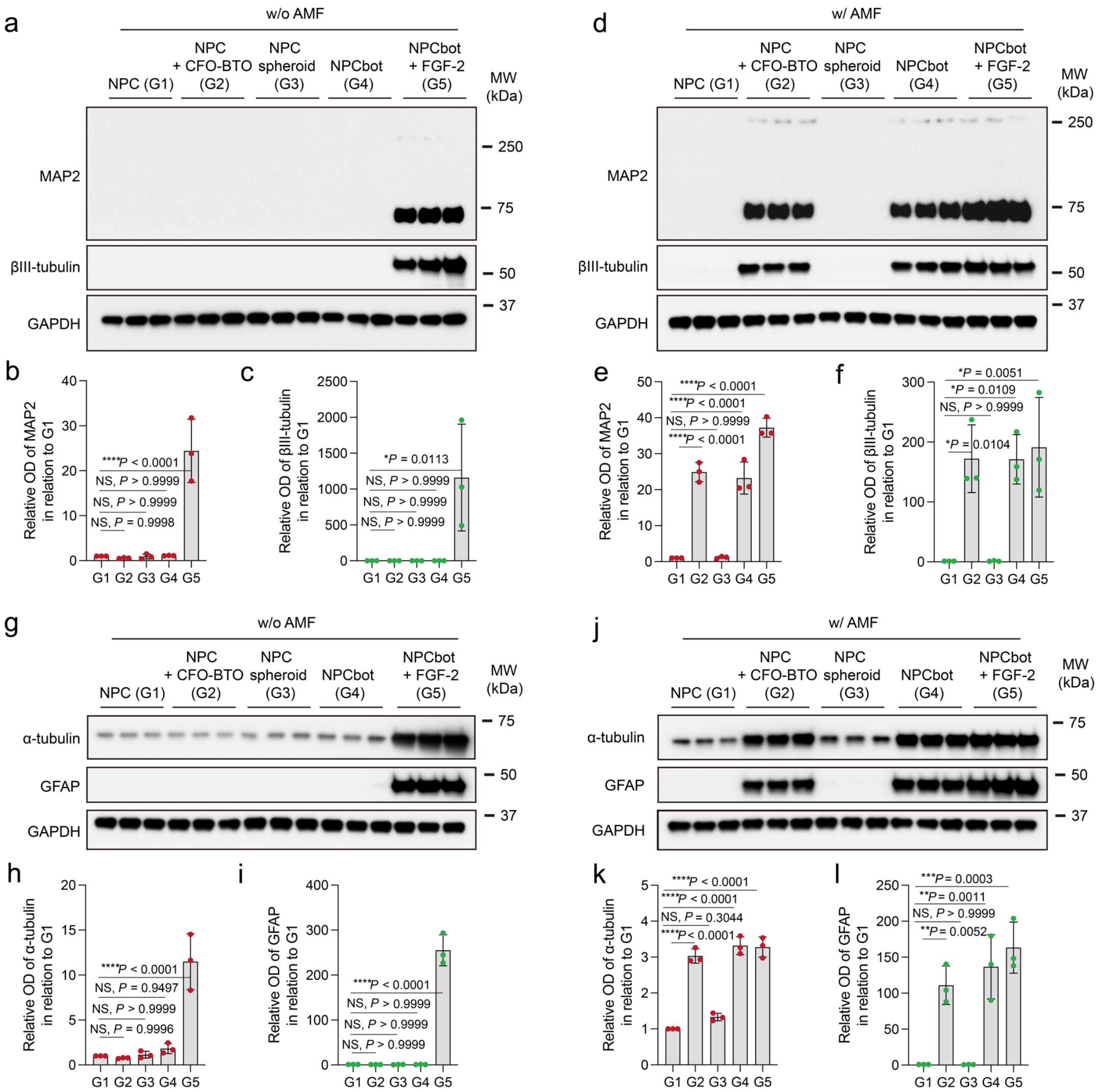

Supplementary Figure 7. Western Blot analysis of NPCbot-induced neural regeneration. a, Western blot assays displaying the expression levels of neuron-specific markers. **b**, Relative quantification of MAP2 and (**c**) βIII-tubulin expression following various treatments (n = 3 experiments, data are presented as mean ± s.d.). **d-f**, Western blot analysis of MAP2 and βIII-tubulin expression under AMF after different treatments (n = 3 experiments, data are presented as mean ± s.d.). **g-i**, Western blot assays detailing the expression levels of the neurite marker α-tubulin and the astrocytic marker GFAP following various treatments (n = 3 experiments, data are presented as mean ± s.d.). **j-l**, Quantification of α-tubulin and GFAP expression under AMF post-treatment (n = 3 experiments, data are presented as mean ± s.d.). Statistical significance was calculated via one-way ANOVA with a Tukey post hoc test.

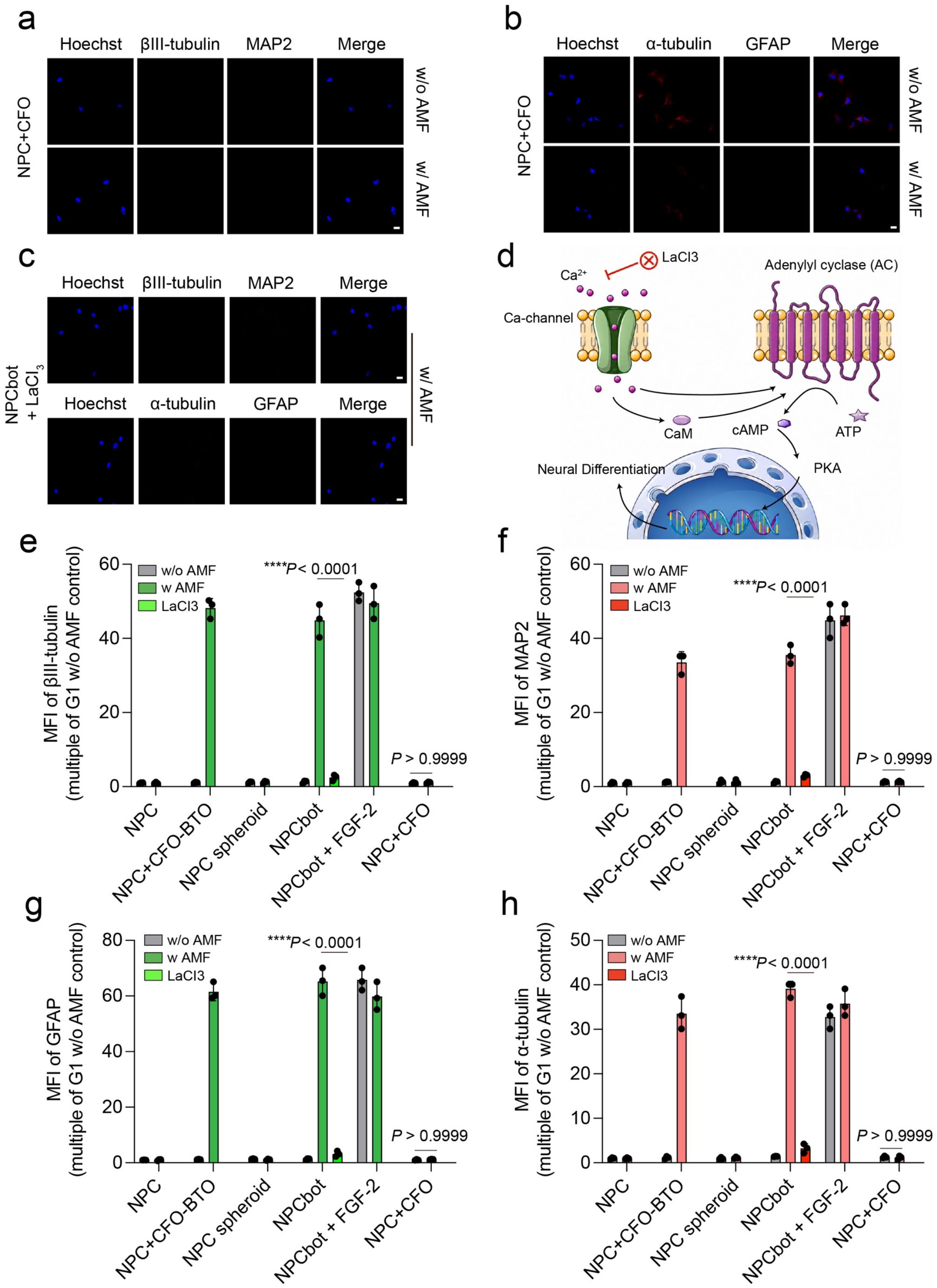

Supplementary Figure 8. Differentiation mechanism analysis. a,b, Immunofluorescence analysis of CFO-treated samples stained for βIII-tubulin and MAP2 or for α-tubulin and GFAP, respectively (Scale bar = 8 μm). **c,** Immunofluorescence analysis of NPCbots stimulated by AMF in the presence of $LaCl_3$ (Scale bar = 8 μm). **d,** Schematic depiction of the intracellular pathways implicated in NPC differentiation influenced by NPCbots and external AMF stimulation. **e-h**, Quantitative assessment through mean fluorescence intensity (MFI) across all samples based on the immunofluorescence results. n = 3 experiments, data are presented as mean± s.d. Statistical significance was calculated via one-way ANOVA with a Tukey post hoc test.

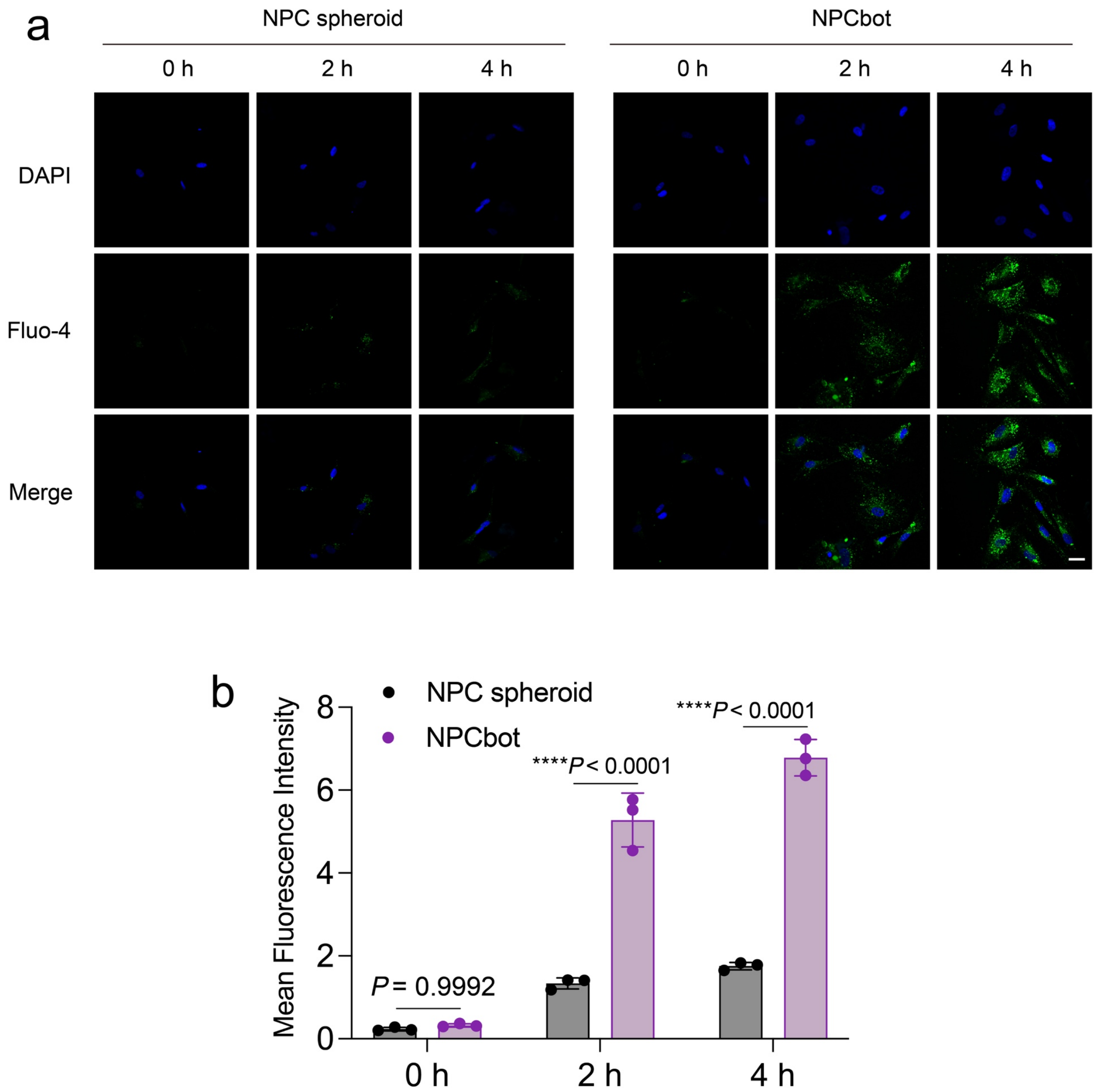

Supplementary Figure 9. Calcium influx. a, Confocal microscopy images showcasing NPC spheroids or NPCbots following AMF stimulation for either 2 h or 4 h, subsequently stained with Fluo-4 AM to indicate intracellular calcium levels (Scale bar = 8 μm). **b**, Quantitative assessment through mean fluorescence intensity (MFI) based on the fluorescence results. n = 3 experiments, data are presented as mean ± s.d. Statistical significance was calculated via one-way ANOVA with a Tukey post hoc test.

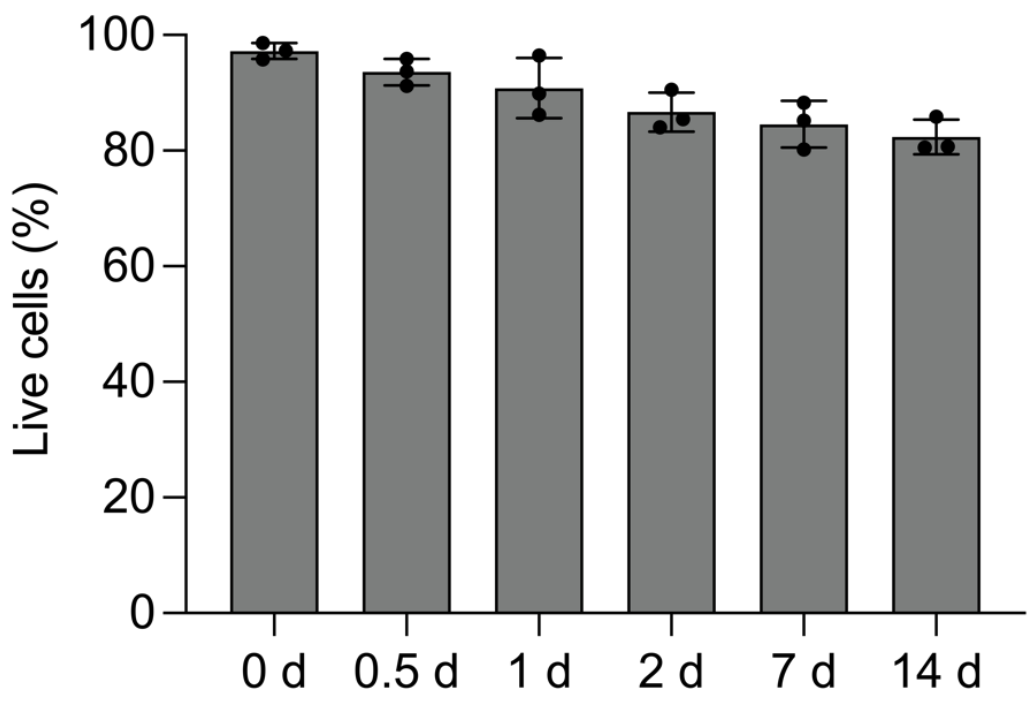

Supplementary Figure 10. Biocompatibility assessment of NPCbots over 14 d AMF stimulation by MTT assay. Data are presented as mean ± s.d, n = 3 experiments.

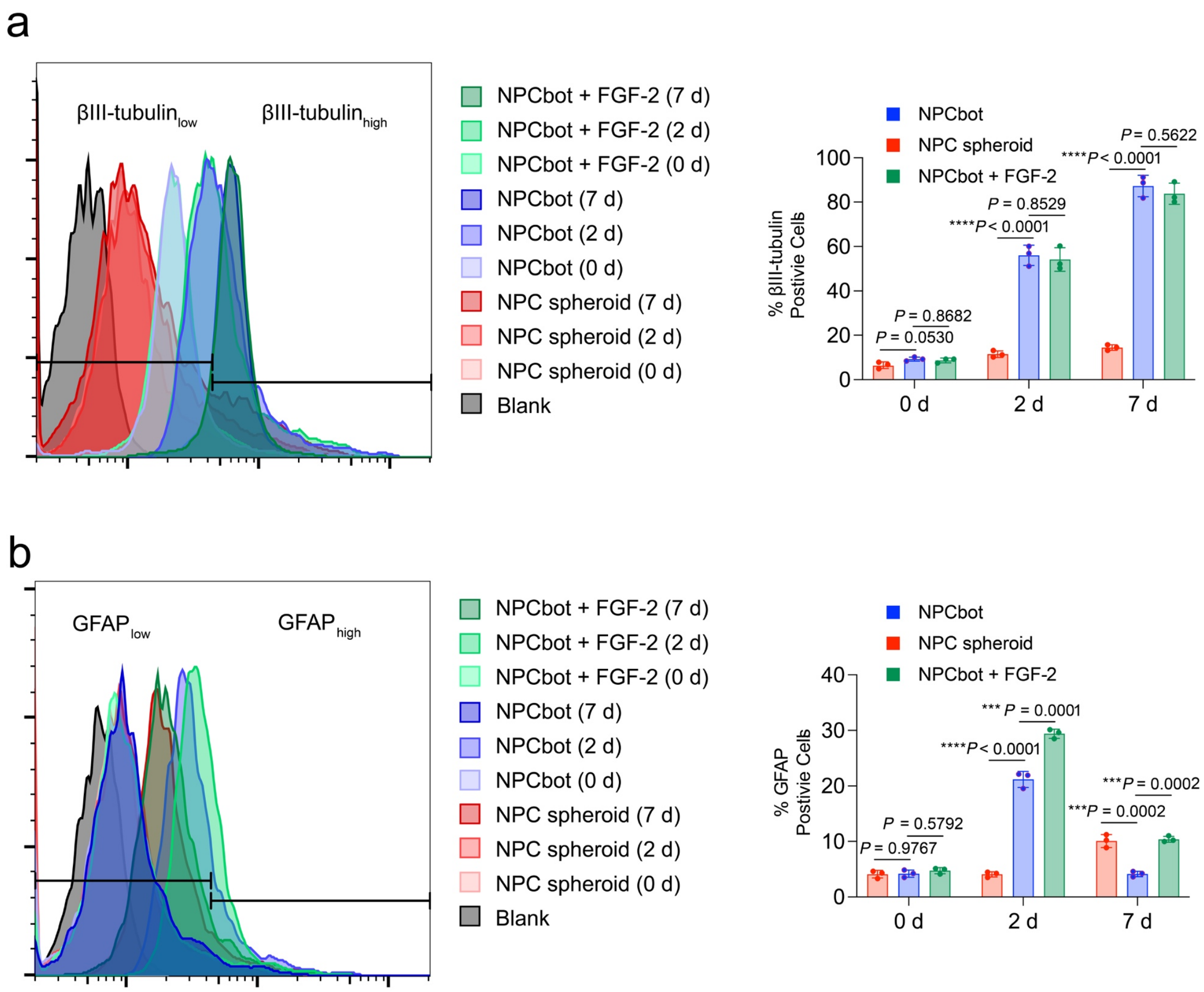

Supplementary Figure 11. Differentiation of NPCbots into Neuronal and Astrocytic Lineages. a, Flow cytometry histograms and quantification of NPCbots stained with Alexa Fluor 488-conjugated βIII-tubulin antibody after 0, 2 and 7 d of stimulation administered twice daily. **b**, GFAP expression in identical experimental conditions, using Alexa Fluor 488-conjugated GFAP antibody. Statistical significance was calculated via one-way ANOVA with a Tukey post hoc test. Data are presented as mean ± s.d, n = 3 experiments.

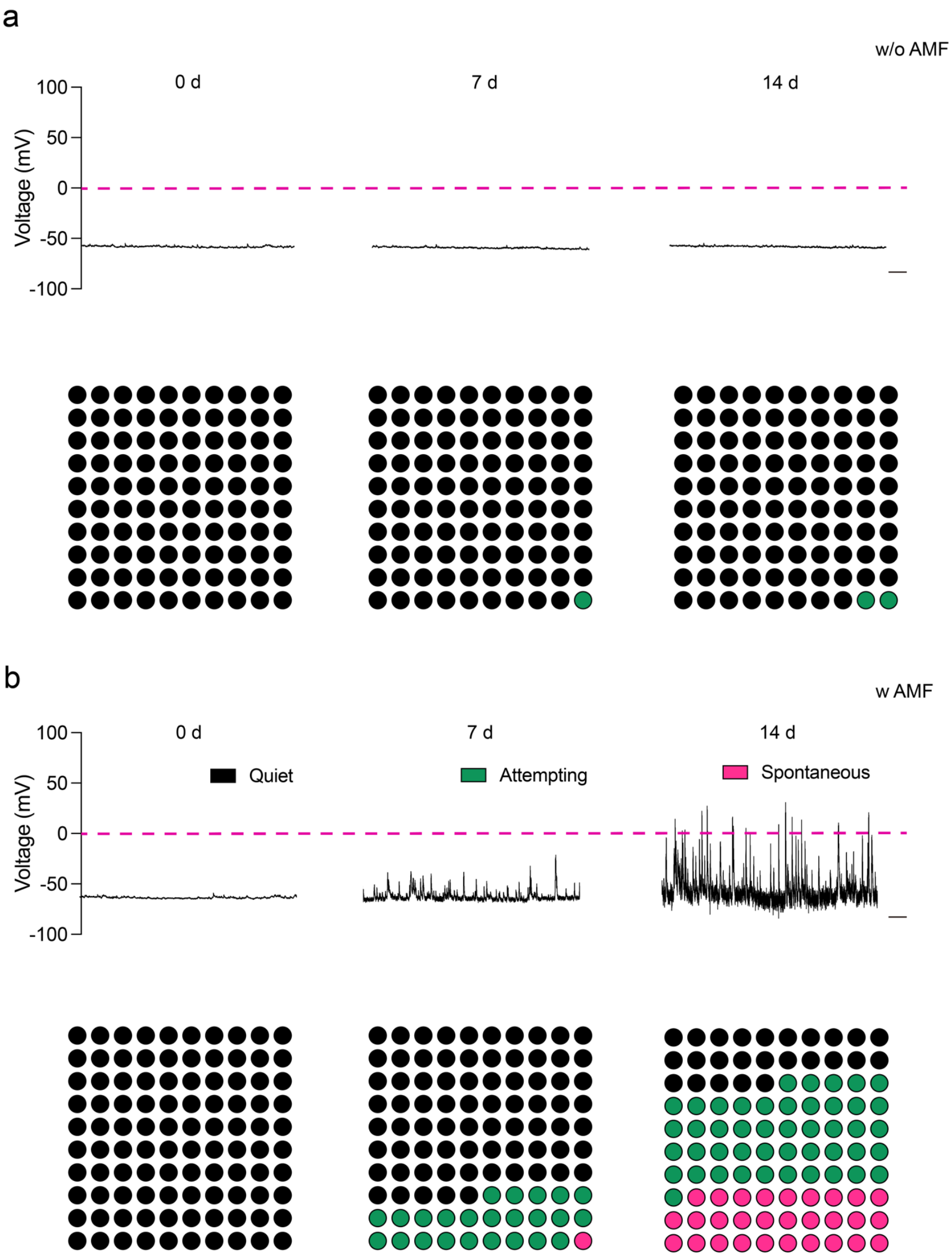

Supplementary Figure 12. Electrophysiological Responses of NPCbots under AMF Stimulation. a, Voltage traces for samples over 0, 7, and 14 days without AMF stimulation. **b**, Electrophysiological recordings from samples subjected to AMF stimulation for 7 and 14 days. The emergence of different firing patterns by patch-clamp recording (quiet, attempting (spontaneous action potential-like oscillations below 0 mV), and spontaneous (action potential)) is summarized by the 10×10 grids below each trace, with the state of 100 individual NPCbots per condition. Scale bar = 400 ms.

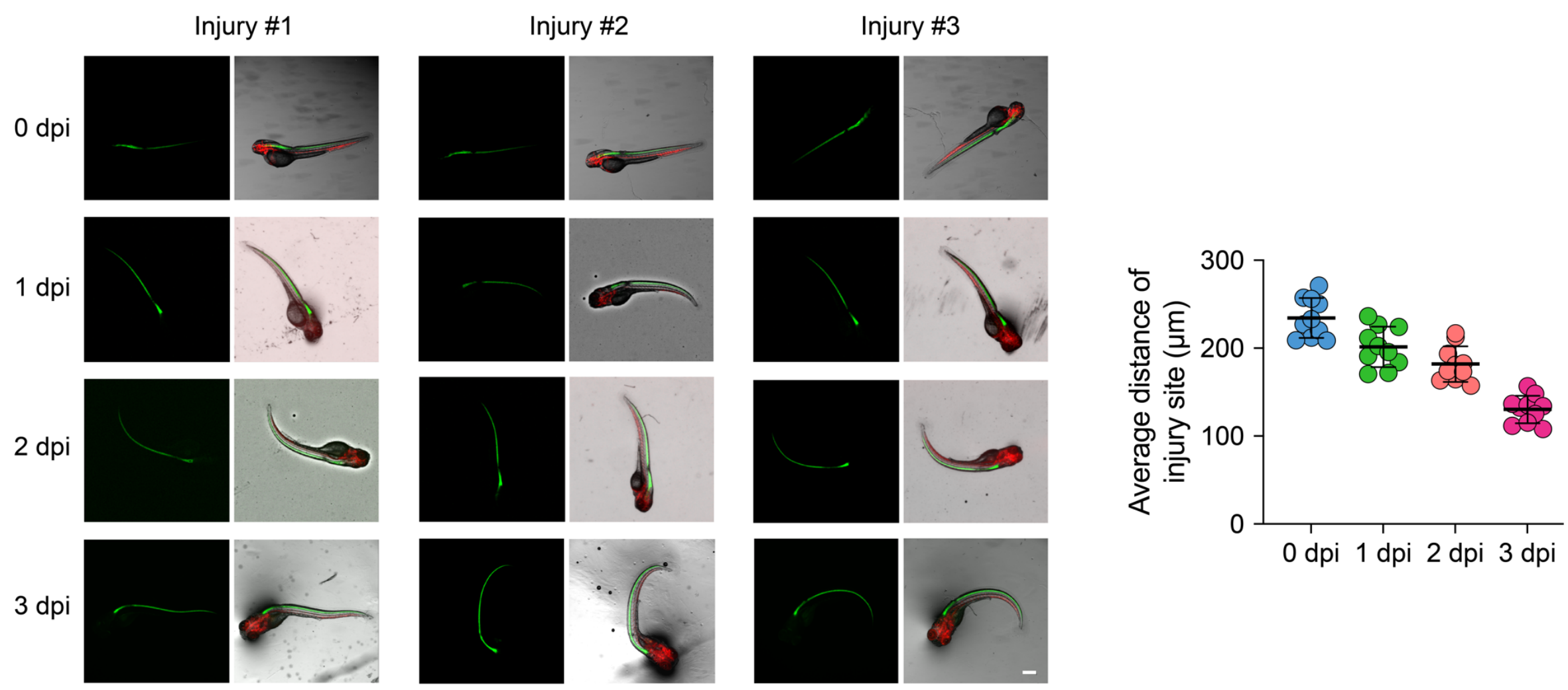

Supplementary Figure 13. Progression of Spinal Cord Regeneration in Zebrafish Larvae Post-Injury. Representative stereomicroscope live imaging of zebrafish larvae following spinal cord injury from 0 to 3 dpi. The graph on the right quantifies the average distance between injury ends, demonstrating the dynamic process of spinal cord regeneration in zebrafish larvae (Data are presented as mean ± s.d, n = 10 larvae, Scale bar = 700 μm).

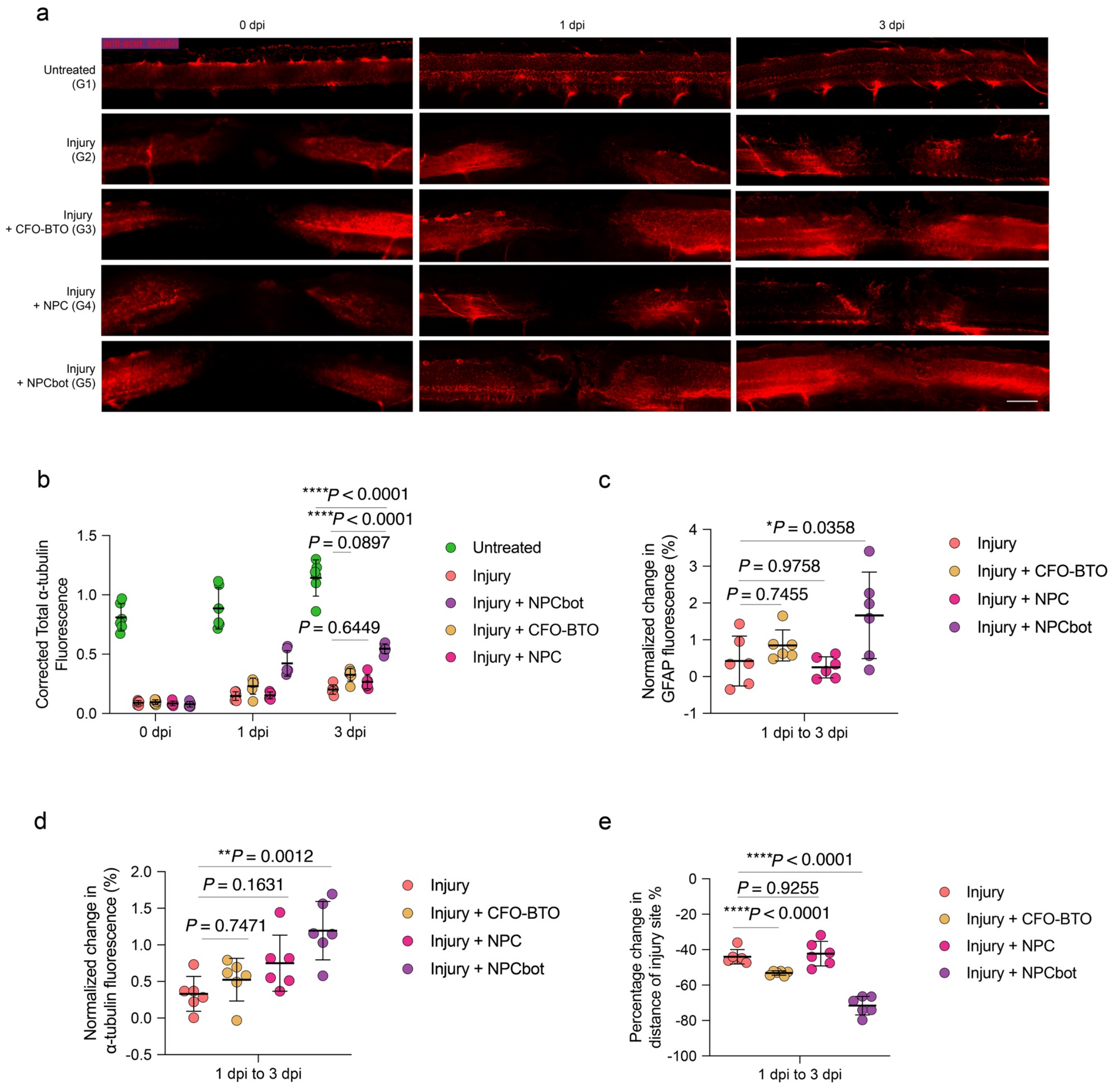

Supplementary Figure 14. Therapeutic efficacy of NPCbots in treating zebrafish spinal cord injuries. **a**, The α-tubulin expression channel corresponding to Fig. 4b, scale bar = 50 μm. **b**, Quantitative analysis of the corrected total intensity of α-tubulin in various experimental groups at 0, 1 and 3 dpi (n = 6 per group, data are presented as mean ± s.d.). **c**, Normalized percentage change in GFAP or (**d**) α-Tubulin in various experimental groups from 1dpi to 3 dpi (n = 6 per group, data are presented as mean ± s.d.). **e**, Percentage change in distance from the injury site in various experimental groups from 1 dpi to 3 dpi (n = 6 per group, data are presented as mean ± s.d.). Statistical significance was calculated via one-way ANOVA with a Tukey post hoc test.

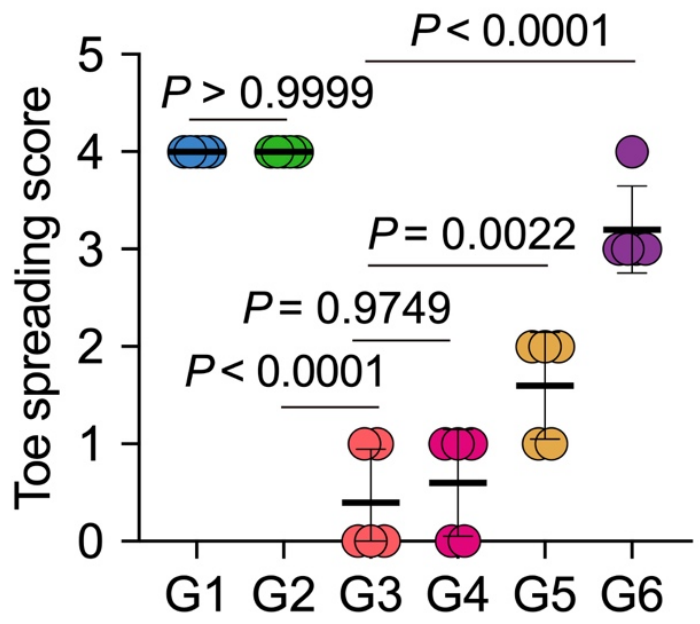

Supplementary Figure 15. Toe spreading score of different groups of mice at day 34. n = 5 per group. Data are presented as mean ± s.d. Statistical significance was calculated via one-way ANOVA with a Tukey post hoc test. G1: Sham; G2: Sham_w/AMF; G3: Injury_w/AMF; G4: Injury + NPC_w/AMF; G5: Injury + CFO-BTO_w/AMF; G6: Injury + NPCbot_w/AMF.

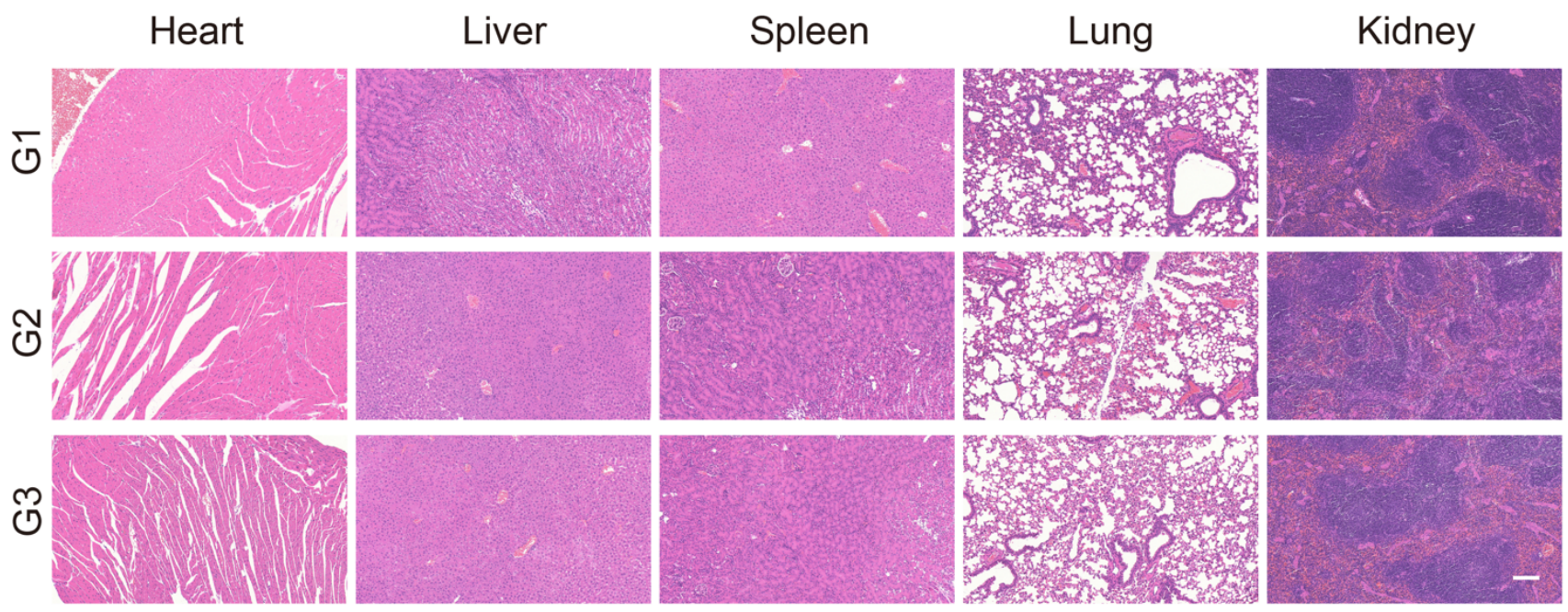

Supplementary Figure 16. H&E staining for major organs at day 35. G1: Sham; G2: Sham_w/AMF; G3: Injury_w/AMF. n = 5 per group, scale bar = 50 μm.

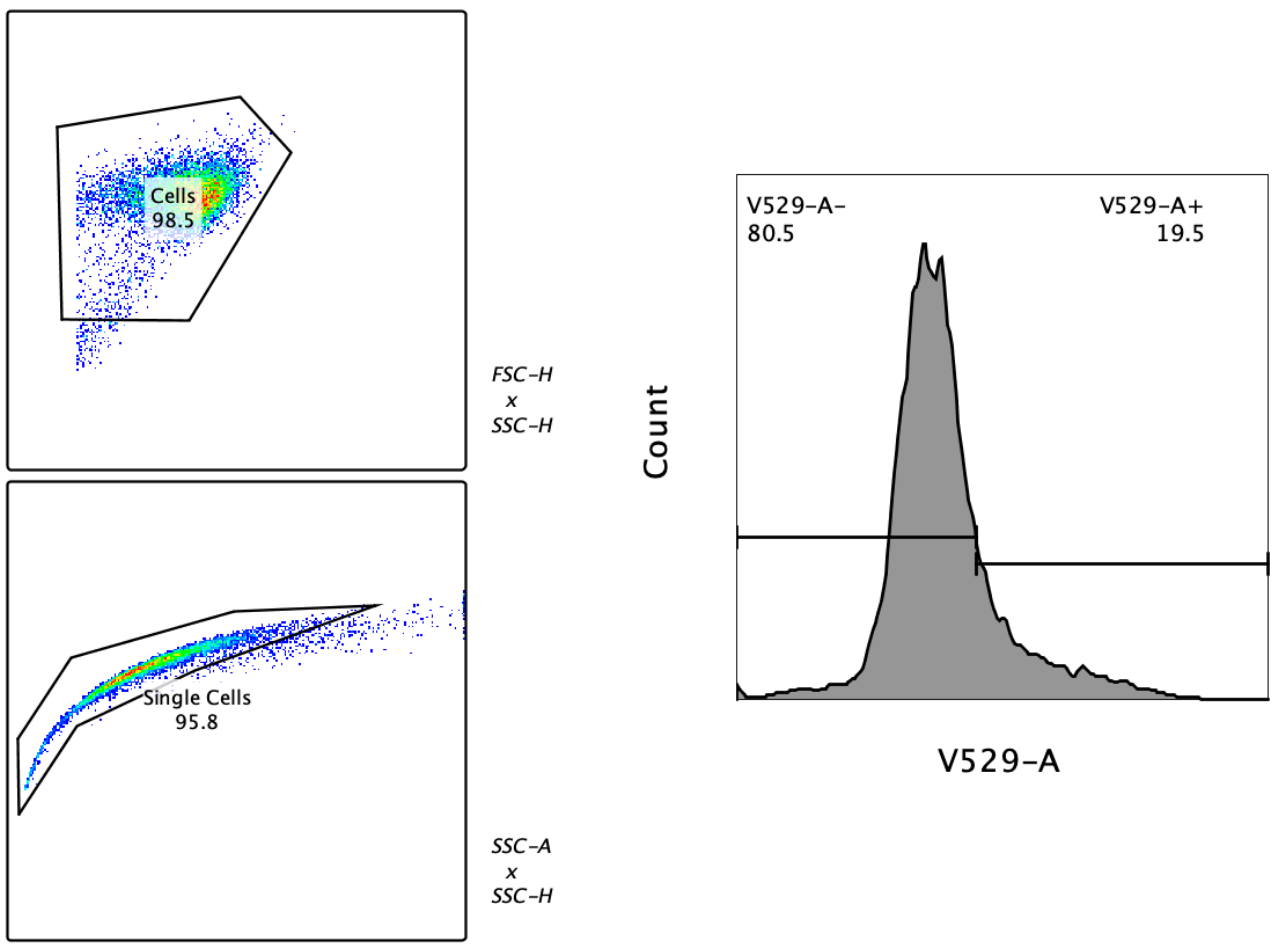

Supplementary Figure 17. Representative flow cytometry gating strategy used to identify marker-positive NPCbots.

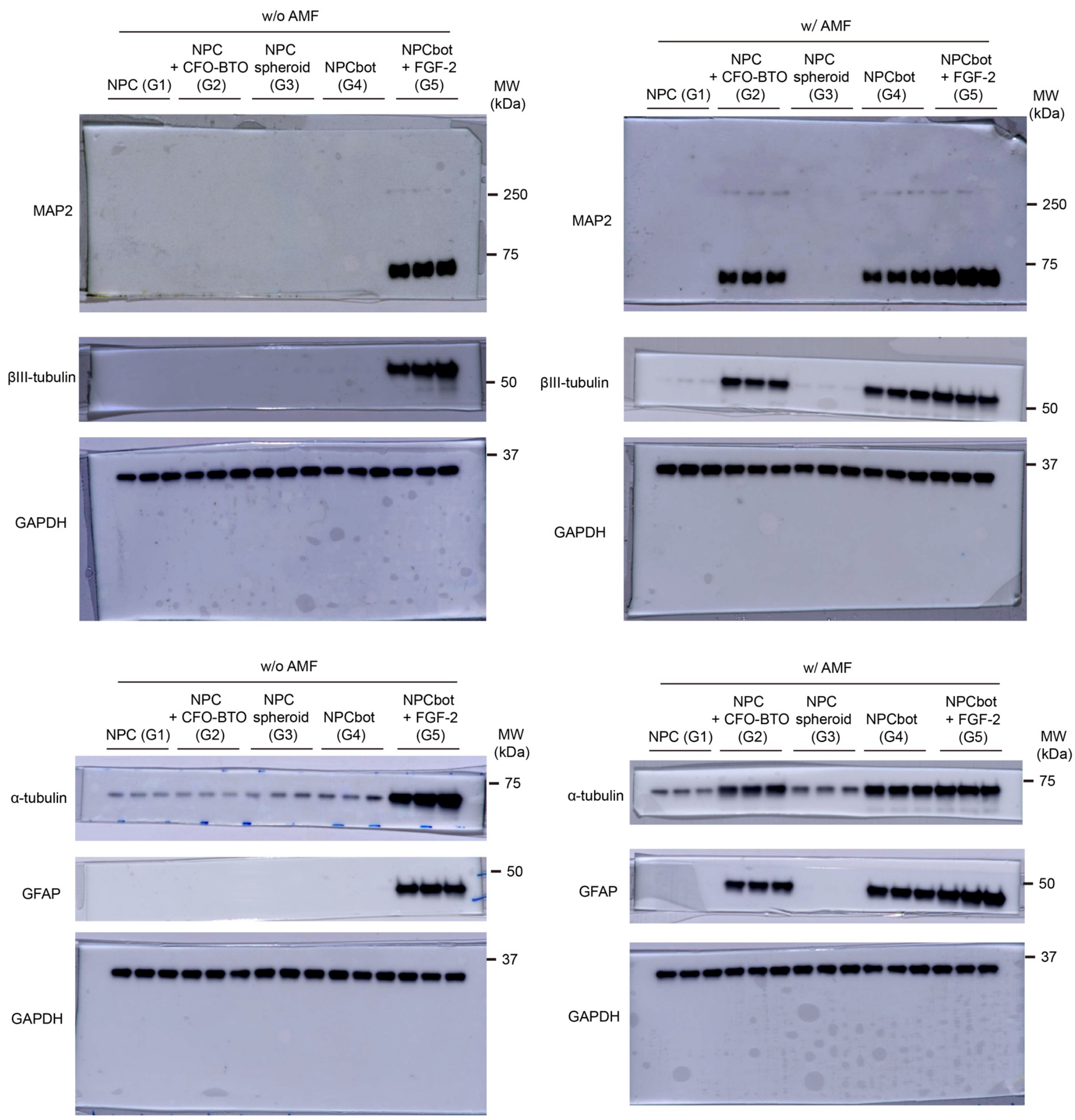

Supplementary Figure 18. Unprocessed western blots corresponding to Supplementary Figure 7.

Supplementary References

1. Kim, D. et al. Magnetoelectric Effect in Hydrogen Harvesting: Magnetic Field as a Trigger of Catalytic Reactions. *Adv Mater* **34**, e2110612 (2022).
2. Stringer, C., Wang, T., Michaelos, M. & Pachitariu, M. Cellpose: a generalist algorithm for cellular segmentation. *Nat Methods* **18**, 100-106 (2021).
3. Kemp, P.J. et al. Improving and accelerating the differentiation and functional maturation of human stem cell-derived neurons: role of extracellular calcium and GABA. *J Physiol* **594**, 6583-6594 (2016).
4. Amores, A. et al. Zebrafish hox clusters and vertebrate genome evolution. *Science* **282**, 1711-1714 (1998).
5. Proulx, K., Lu, A. & Sumanas, S. Cranial vasculature in zebrafish forms by angioblast cluster-derived angiogenesis. *Dev Biol* **348**, 34-46 (2010).
6. Bernardos, R.L. & Raymond, P.A. GFAP transgenic zebrafish. *Gene Expr Patterns* **6**, 1007-1013 (2006).
7. John, N., Kolb, J. & Wehner, D. Mechanical spinal cord transection in larval zebrafish and subsequent whole-mount histological processing. *STAR Protoc* **3**, 101093 (2022).
