# Supplementary material for: Magnetoelectric microrobots for spinal cord injury regeneration
Source: Nat Mater. 2026 Jun 2;25(8):1458–69. doi: 10.1038/s41563-026-02625-3 (PMC13658017; doi:10.1038/s41563-026-02625-3)
Supplement: Supplementary file 2 [file 41563_2026_2625_MOESM2_ESM.pdf]

nature portfolio

Corresponding author(s): Jingjing Zang; Semih Sevim; Xiang-Zhong Chen; Cong Luo; Bradley J. Nelson; Stephan C. F. Neuhauss; Salvador Pané

Last updated by author(s): Apr 26, 2026

Reporting Summary

Nature Portfolio wishes to improve the reproducibility of the work that we publish. This form provides structure for consistency and transparency in reporting. For further information on Nature Portfolio policies, see our Editorial Policies and the Editorial Policy Checklist.

Statistics

For all statistical analyses, confirm that the following items are present in the figure legend, table legend, main text, or Methods section.

n/a	Confirmed	
☐	☒	The exact sample size (*n*) for each experimental group/condition, given as a discrete number and unit of measurement
☐	☒	A statement on whether measurements were taken from distinct samples or whether the same sample was measured repeatedly
☐	☒	The statistical test(s) used AND whether they are one- or two-sided<br>*Only common tests should be described solely by name; describe more complex techniques in the Methods section.*
☒	☐	A description of all covariates tested
☐	☒	A description of any assumptions or corrections, such as tests of normality and adjustment for multiple comparisons
☐	☒	A full description of the statistical parameters including central tendency (e.g. means) or other basic estimates (e.g. regression coefficient) AND variation (e.g. standard deviation) or associated estimates of uncertainty (e.g. confidence intervals)
☐	☒	For null hypothesis testing, the test statistic (e.g. *F*, *t*, *r*) with confidence intervals, effect sizes, degrees of freedom and *P* value noted<br>*Give P values as exact values whenever suitable.*
☒	☐	For Bayesian analysis, information on the choice of priors and Markov chain Monte Carlo settings
☒	☐	For hierarchical and complex designs, identification of the appropriate level for tests and full reporting of outcomes
☒	☐	Estimates of effect sizes (e.g. Cohen's *d*, Pearson's *r*), indicating how they were calculated

Our web collection on statistics for biologists contains articles on many of the points above.

Software and code

Policy information about availability of computer code

Data collection	Zeiss Zen (3.4);<br>Leica Application Suite X (3.5.7.23225);<br>BD FACSDivaTM (9.0.1);<br>Zebralab (3.10)<br>Clampex (10.2)<br>cellSens (3.2.1)<br>Imagelab (6.1)
Data analysis	GraphPad Prism (9.5.0)<br>Imaris (9.9.1)<br>ImageJ (2.3.0)<br>EthoVision XT (11.5)<br>ImageQuant TL (8.1)<br>Heml (1.0.3.7)

For manuscripts utilizing custom algorithms or software that are central to the research but not yet described in published literature, software must be made available to editors and reviewers. We strongly encourage code deposition in a community repository (e.g. GitHub). See the Nature Portfolio guidelines for submitting code & software for further information.

Data

Policy information about availability of data

All manuscripts must include a data availability statement. This statement should provide the following information, where applicable:

- Accession codes, unique identifiers, or web links for publicly available datasets
- A description of any restrictions on data availability
- For clinical datasets or third party data, please ensure that the statement adheres to our policy

Source data are provided with this paper, and all relevant data are available in the main text, Supplementary Information, Source Data and Supplementary Data files.

Research involving human participants, their data, or biological material

Policy information about studies with human participants or human data. See also policy information about sex, gender (identity/presentation), and sexual orientation and race, ethnicity and racism.

Reporting on sex and gender	N/A
Reporting on race, ethnicity, or other socially relevant groupings	N/A
Population characteristics	N/A
Recruitment	N/A
Ethics oversight	N/A

Note that full information on the approval of the study protocol must also be provided in the manuscript.

Field-specific reporting

Please select the one below that is the best fit for your research. If you are not sure, read the appropriate sections before making your selection.

☒ Life sciences ☐ Behavioural & social sciences ☐ Ecological, evolutionary & environmental sciences

For a reference copy of the document with all sections, see nature.com/documents/nr-reporting-summary-flat.pdf

Life sciences study design

All studies must disclose on these points even when the disclosure is negative.

Sample size	No statistical methods were used to predetermine the sample sizes, but our sample sizes are similar to those reported in previous publications.
Data exclusions	No data were excluded.
Replication	All experiments were repeated at least 3 times and the experimental results are reproducible.
Randomization	Samples/organisms were allocated into groups at random.
Blinding	For all animal studies, subjects were randomly assigned to experimental groups. BMS scores were evaluated by independent observers blinded to the treatment conditions. While blinding was not feasible during the execution of in vitro studies due to procedural requirements, data analysis remained unbiased. All quantitative assessments were performed blinded to group allocation, with samples only being re-identified for final visualization and statistical testing.

Reporting for specific materials, systems and methods

We require information from authors about some types of materials, experimental systems and methods used in many studies. Here, indicate whether each material, system or method listed is relevant to your study. If you are not sure if a list item applies to your research, read the appropriate section before selecting a response.

Materials & experimental systems

n/a	Involved in the study
☐	☒ Antibodies
☐	☒ Eukaryotic cell lines
☒	☐ Palaeontology and archaeology
☐	☒ Animals and other organisms
☒	☐ Clinical data
☒	☐ Dual use research of concern
☒	☐ Plants

Methods

n/a	Involved in the study
☒	☐ ChIP-seq
☐	☒ Flow cytometry
☒	☐ MRI-based neuroimaging

Antibodies

Antibodies used

Primary antibodies: anti-βIII-tubulin antibody (Merck, T2200, 1:100), MAP2 monoclonal antibody (Proteintech, 67015-1, 1:100), monoclonal anti-tubulin, acetylated antibody produced in mouse (Merck, T6793, 1:300), glial fibrillary acidic protein (Agilent, Z0334, 1:300), anti-Nestin, human antibody (Sigma, ABD69, 1:400), anti-SOX2 antibody (Sigma, MAB4343, 1:100), anti-βIII-tubulin (Abcam, ab18207, 1:1000), anti-α-tubulin (Abcam, ab52866, 1:1000), anti-GFAP (Abcam, ab68428, 1:10000), anti-MAP2 (Abcam, ab32454, 1:1000), βIII-tubulin (Abcam, ab78078, 1:1000), GFAP (Abcam, ab7260, 1:1000)

Secondary antibodies: FITC-conjugated goat anti-rabbit IgG (Sigma-Aldrich, AP132F, 1:200), TRITC-conjugated goat anti-mouse IgG (Sigma-Aldrich, AP503R, 1:200), HRP-conjugated Affinipure goat anti-rabbit IgG (H+L) (Proteintech, SA00001-2, 1:10000), Alexa Fluor 488 anti-rabbit (Thermo Fisher Scientific, Cat#A32731, 1:300), Alexa Fluor 555 anti-mouse (Thermo Fisher Scientific, Cat#A11008, 1:300), goat anti-mouse IgG H&L conjugated with Alexa Fluor 647 (Abcam, ab150115, 1:1000), and goat anti-rabbit IgG H&L conjugated with Cy3 (Abcam, ab6939, 1:1000).

Flow cytometry antibodies: Alexa Fluor 488 βIII-tubulin antibody (BioLegend, Cat. No. 801203, 1:20), Alexa Fluor 488 GFAP antibody (BD Biosciences, Cat. No. 561449, 1:20).

Validation

All antibodies were validated by the suppliers, and each lot was quality-tested. The corresponding validation information is available on the respective antibody product webpages.

Anti-βIII-tubulin: https://www.sigmaaldrich.com/CH/fr/product/sigma/t2200?srsltid=AfmBOoqKdjSlqMKN6oHt-UWF-SzE_3uB7iq8C-jas9Amke1eIWf5mMzR
Anti-MAP2: https://www.ptglab.com/products/MAP2-Antibody-67015-1-Ig.htm?srsltid=AfmBOorgwIEdXBh917qNQU1ylOGFrbmO5P08AMkLHTPOzl-jjqItJqfL
Anti-α-tubulin: https://www.sigmaaldrich.com/CH/en/product/sigma/t6793
Anti-GFAP: https://www.agilent.com/en/product/immunohistochemistry/antibodies-controls/primary-antibodies/glial-fibrillary-acidic-protein-(concentrate)-76683
Anti-Nestin: https://www.sigmaaldrich.com/CH/en/product/mm/abd69?srsltid=AfmBOooUiRxHX8Y5osOyUbkJXqxzigtIDVREf5KHxevX5p5GLkrf5b84
Anti-SOX2: https://www.sigmaaldrich.com/CH/en/product/mm/mab4343?srsltid=AfmBOoptX37ZCzqXecCgkDF6u4ikqEB06m3Lfq-X13c0qYOaG6imSlE4
Anti-βIII-tubulin: https://www.abcam.com/en-us/products/primary-antibodies/beta-iii-tubulin-antibody-neuronal-marker-ab18207
Anti-α-tubulin: https://www.abcam.com/en-us/products/primary-antibodies/alpha-tubulin-antibody-ep1332y-loading-control-ab52866
Anti-GFAP: https://www.abcam.com/en-us/products/primary-antibodies/gfap-antibody-epr1034y-astrocyte-marker-ab68428
Anti-MAP2: https://www.abcam.com/en-us/products/primary-antibodies/map2-antibody-neuronal-marker-ab32454
Anti-βIII-tubulin: https://www.abcam.com/en-us/products/primary-antibodies/beta-iii-tubulin-antibody-2g10-neuronal-marker-ab78078
Anti-GFAP: https://www.abcam.com/en-us/products/primary-antibodies/gfap-antibody-astrocyte-marker-ab7260

Eukaryotic cell lines

Policy information about cell lines and Sex and Gender in Research

Cell line source(s)

Human iPSC Derived Neural Progenitors (Cat. No. SCC035, Merck), mouse spinal cord neural stem cells (Cat. No. SCR031, Merck).

Authentication

Cell identity and neural progenitor phenotype were verified by supplier documentation and by in-house immunofluorescence and flow cytometry analysis of Nestin and SOX2 expression.

Mycoplasma contamination

The cells were tested for mycoplasma contamination, and no evidence of contamination was detected.

Commonly misidentified lines (See ICLAC register)

No commonly misidentified lines were used in this study.

Animals and other research organisms

Policy information about studies involving animals; ARRIVE guidelines recommended for reporting animal research, and Sex and Gender in Research

Laboratory animals	8-week-old male C57BL/6 mice (18-22g) were purchased from Liaoning Changsheng Biotechnology Co., Ltd. and housed at the Animal Center of Shenyang Pharmaceutical University, China. The animals were maintained in a room with air humidity ranging from 40% to 70%, a temperature of 21-23 °C, and a 12-hour dark/light cycle.
Wild animals	No wild animals were used in this study.
Reporting on sex	Male mice were used to establish a spinal cord injury model in this study.
Field-collected samples	No field-collected samples were used in this study.
Ethics oversight	All experimental procedures were conducted in strict compliance with the guidelines approved by the Institutional Animal Ethical Care Committee (IAEC) of Shenyang Pharmaceutical University (approval number: SYXK 2021-0009, Liaoning, China). Zebrafish embryos and larvae were used at 2–5 dpf, before independent feeding. Therefore, these experiments did not require animal experimentation approval under the applicable regulatory framework. Zebrafish were maintained under standard laboratory conditions as described in the Methods.

Note that full information on the approval of the study protocol must also be provided in the manuscript.

Plants

Seed stocks	N/A
Novel plant genotypes	N/A
Authentication	N/A

Flow Cytometry

Plots

Confirm that:

☒ The axis labels state the marker and fluorochrome used (e.g. CD4-FITC).

☒ The axis scales are clearly visible. Include numbers along axes only for bottom left plot of group (a 'group' is an analysis of identical markers).

☒ All plots are contour plots with outliers or pseudocolor plots.

☒ A numerical value for number of cells or percentage (with statistics) is provided.

Methodology

Sample preparation	Cells were minimally processed (trypsinized and resuspended in standard media).
Instrument	BD LSRFortessa
Software	BD FACSDivaTM (9.0.1), Flowjo (10.4).
Cell population abundance	Approximately 10,000 cells were analyzed for their fluorescent intensity within the specified gate.
Gating strategy	Gating was first based on FSC/SSC and single cells were gated for further analysis. The cell populations were then analyzed based on expression of markers. Gating was then based on positive level.

☒ Tick this box to confirm that a figure exemplifying the gating strategy is provided in the Supplementary Information.
